# Structurally reprogrammed modified citrus pectin (MCP) enables potentiated galectin-3 sequestration and injectable carboxymethyl chitosan/berberine hydrogel construction for osteoarthritis immunotherapy

**DOI:** 10.1016/j.mtbio.2025.102330

**Published:** 2025-09-20

**Authors:** Chi Lin, Fwu-Long Mi, Chia-Yun Cha, Fang-Yu Hsu, Siti Ayu Ulfadillah, Min-Lang Tsai, Hsien-Tsung Lu

**Affiliations:** a Department of Biochemistry and Molecular Cell Biology, School of Medicine, College of Medicine, Taipei Medical University, Taipei 11031, Taiwan, ROC; b Graduate Institute of Medical Sciences, College of Medicine, Taipei Medical University, Taipei 11031, Taiwan, ROC; c Graduate Institute of Nanomedicine and Medical Engineering, College of Biomedical Engineering, Taipei Medical University, Taipei 11031, Taiwan, ROC; d International Ph.D. Program in Cell Therapy and Regenerative Medicine, Taipei Medical University, Taipei 11031, Taiwan, ROC; e Department of Food Science, National Taiwan Ocean University, Keelung 202301, Taiwan, ROC; f Sriwijaya University, Department of Fisheries Product Technology, Agriculture Faculty, Indralaya 30662, South Sumatra, Indonesia; g Department of Orthopedics, Taipei Medical University Hospital, Taipei 11031, Taiwan, ROC; h Department of Orthopedics, School of Medicine, College of Medicine, Taipei Medical University, Taipei 11031, Taiwan, ROC

**Keywords:** Osteoarthritis, Modified citrus pectin, Berberine, Injectable hydrogel, Macrophage polarization

## Abstract

Osteoarthritis (OA) remains a major clinical challenge due to the lack of effective treatments capable of halting disease progression. Chronic synovial inflammation and cartilage degradation are hallmark features, wherein galectin-3 (Gal-3), a β-galactoside-binding lectin, plays a pivotal upstream role by driving M1 macrophage activation and chondrocyte apoptosis. Modified citrus pectin (MCP), a natural Gal-3 binder, possesses therapeutic potential but is hindered by rapid clearance and limited joint retention. Herein, we present oxidized MCP (oxMCP), structurally reprogrammed MCP, that functions as a Gal-3-sequestering and crosslinkable matrix. This transformation was achieved via periodate oxidation, which introduced dialdehyde groups for Schiff base crosslinking with N, O-carboxymethyl chitosan (NOCC), while simultaneously enhancing Gal-3 affinity by reducing the molecular weight, increasing the chain flexibility, and exposing β-(1 → 4)-galactan motifs. These changes markedly amplified Gal-3-associated bioactivities, including M1 macrophage suppression and chondroprotection. The resulting oxMCP/NOCC hydrogel was further integrated with berberine (BBR), a cationic alkaloid with M2-polarizing activity, which reinforced the hydrogel network via non-covalent interactions and empowered the M2-polarizing capacity. The oxMCP/NOCC/BBR hydrogel exhibited excellent self-healing, low swelling, slow degradation, and sustained drug release, key features for intra-articular delivery. *In vitro*, it suppressed oxidative stress, matrix degradation, and chondrocyte apoptosis while promoting macrophage polarization toward the M2 phenotype. In vivo, intra-articular administration alleviated synovial inflammation and preserved cartilage in a rat OA model. This work transformed MCP from a short-acting Gal-3 blocker into a durable, bioactivity-enhanced therapeutic platform with immunomodulatory and cartilage-protective capabilities, offering a transformative strategy for a localized pathology-adaptive OA intervention.

## Graphical abstract

**Figure:**
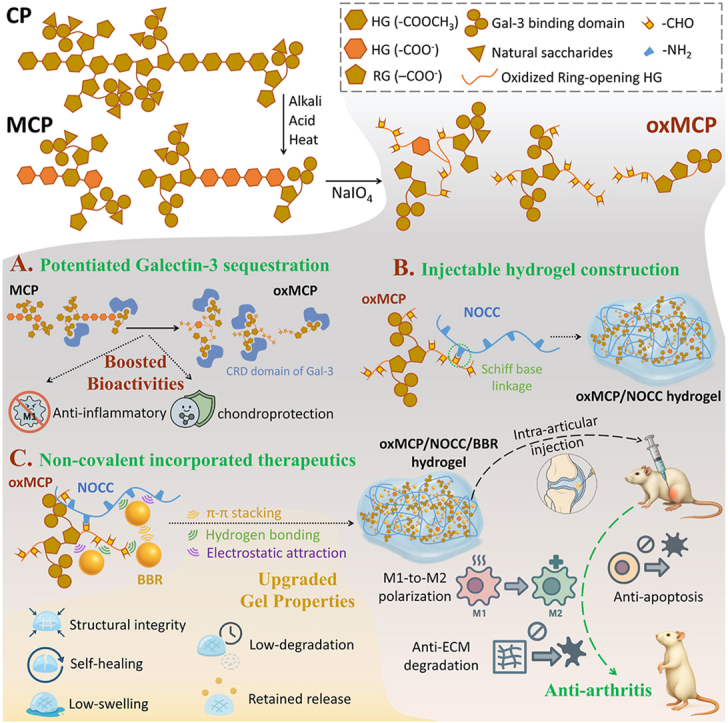

## 1 Introduction

Osteoarthritis (OA) is a chronic, degenerative joint disorder characterized by progressive articular cartilage degradation, synovial inflammation, and subchondral bone remodeling, ultimately leading to chronic pain, impaired mobility, and reduced quality of life [1,2]. A central driver of OA pathogenesis is sustained low-grade inflammation, mediated by pro-inflammatory cytokines such as interleukin-1β (IL-1β), tumor necrosis factor-α (TNF-α), and interleukin-6 (IL-6) [3]. These cytokines stimulate the production of matrix metalloproteinases (MMPs) and aggrecanases while suppressing anabolic signals for type II collagen and proteoglycan synthesis, culminating in chondrocyte apoptosis and matrix breakdown [4]. Although current pharmacologic interventions, such as nonsteroidal anti-inflammatory drugs (NSAIDs), intra-articular corticosteroids, and hyaluronic acid (HA) injections, offer temporary symptomatic relief, they fail to prevent structural joint deterioration and are often associated with a limited duration of efficacy or with systemic side effects [5]. While regenerative approaches including autologous chondrocyte implantation and mesenchymal stem cell (MSC) transplantation have shown potential, their therapeutic outcomes remain inconsistent due to the hostile inflammatory microenvironment in OA joints [6]. Thus, there is an urgent need for disease-modifying strategies capable of both suppressing inflammation and promoting cartilage repair in a sustained and localized manner [[7], [8], [9], [10], [11], [12]].

Galectin-3 (Gal-3), a β-galactoside-binding lectin, has emerged as a key upstream mediator of OA pathophysiology [[13], [14], [15]]. It is highly expressed by OA cartilage and synovial tissue, where it promotes pro-inflammatory macrophage polarization, chondrocyte apoptosis, and matrix degradation. Targeting Gal-3 has therefore gained attention as a promising immunomodulatory strategy for OA therapy. Modified citrus pectin (MCP), a low-molecular-weight polysaccharide derived from the alkaline and acidic treatment of citrus pectin (CP), is enriched in β-galactose residues that bind with high affinity to the carbohydrate recognition domain (CRD) of Gal-3 [13,14,16]. Through this interaction, MCP has demonstrated anti-inflammatory, chondroprotective, and chondroproliferative effects in preclinical models by sequestering Gal-3 and attenuating its pathological activity in joint tissues [13,14,17]. However, its therapeutic efficacy remains limited due to poor tissue retention and rapid clearance following intra-articular administration [13,14]. To address these limitations, formulating MCP as a primary structural component of a crosslinked hydrogel matrix may offer a means to prolong its intra-articular retention.

To this end, MCP was chemically engineered into a dialdehyde-functionalized form, oxidized MCP (oxMCP), via periodate-mediated oxidation [18,19], thereby enabling dynamic Schiff base crosslinking with amino-bearing polymers such as N, O-carboxymethyl chitosan (NOCC), a biocompatible, amino-rich polysaccharide that is structurally similar to HA [20]. This chemical modification was intended not only to support hydrogel formation through reversible covalent bonding, but also to ensure injectability and shape adaptability, hallmark features of Schiff base hydrogels [[21], [22], [23]], suitable for intra-articular use. In parallel, such structural modifications may contribute to enhanced Gal-3-associated biological activities of MCP, such as chondroprotection and immunomodulatory effects, by reducing its molecular weight, increasing backbone flexibility, and exposing Gal-3-binding sugar domains, including β-(1 → 4)-galactan residues known to play a critical role in lectin recognition [19,[24], [25], [26]]. Specifically, periodate-induced ring opening disrupts the rigid pyranose structure and partially removes branched side chains, while periodate-derived radicals may contribute to a decrease in molecular weight. These structural changes may collectively enhance the accessibility of β-(1 → 4)-galactan residues within MCP to the CRD of Gal-3.

Functional hydrogel systems co-loading bioactive molecules have also been explored to enhance therapeutic performance and stability [27]. Promoting M1-to-M2 macrophage polarization has emerged as a critical strategy in tissue engineering to resolve chronic inflammation and support tissue regeneration [28]. Berberine (BBR), a plant-derived isoquinoline alkaloid with well-documented anti-inflammatory, antioxidant, and chondroprotective activities [[29], [30], [31], [32], [33]], was incorporated into the oxMCP/NOCC hydrogel to complement the Gal-3-directed anti-inflammatory action of oxMCP and endow the system with a robust immunoreprogramming capacity, particularly by promoting M1-to-M2 macrophage polarization. Structurally, BBR carries a permanent positive charge due to its quaternary ammonium group and possesses multiple aromatic and polar functional moieties [20]. These features were expected to promote electrostatic interactions with the negatively charged carboxyl groups of oxMCP, as well as hydrogen bonding and π–π stacking within the hydrogel network. Such non-covalent interactions were anticipated to enhance network cohesion, improve structural stability, and contribute to a more sustained and controllable release of BBR at the target site [20].

**Fig. 1:**
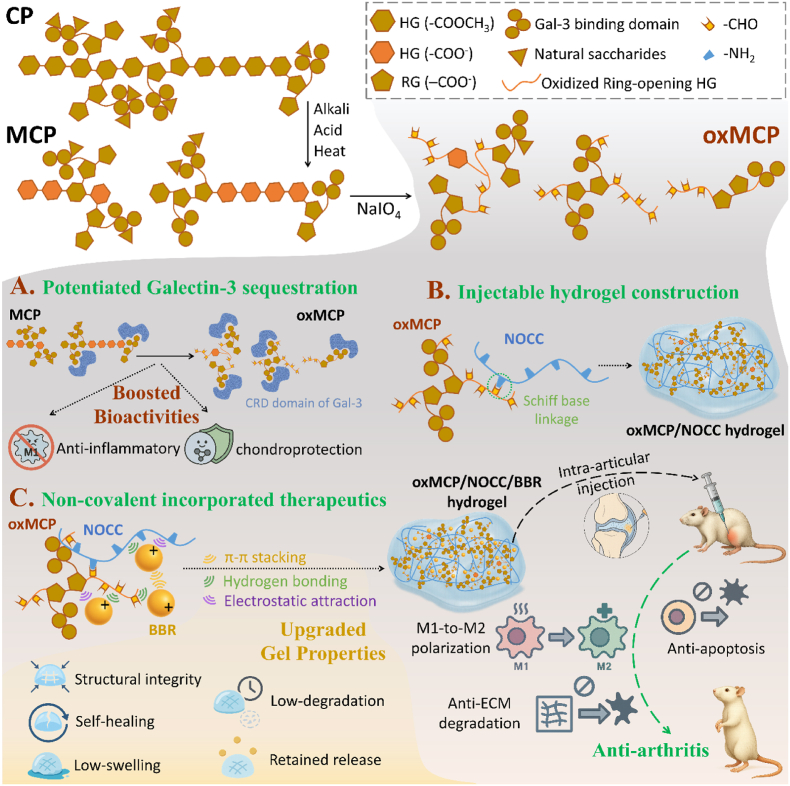
Schematic illustration of the design and function of the oxMCP/NOCC/BBR hydrogel platform for intra-articular osteoarthritis therapy. The structurally reprogrammed oxMCP was prepared via sequential alkali–acid–heat treatment and NaIO_4_-mediated oxidation to generate an aldehyde-functionalized backbone with enhanced exposure of Gal-3-binding domains. (A) oxMCP exhibited an increased Gal-3 binding affinity compared to MCP, leading to potentiated anti-inflammatory and chondroprotective bioactivities. (B) oxMCP formed an injectable hydrogel via dynamic Schiff base crosslinking with NOCC. (C) BBR was incorporated via non-covalent interactions to reinforce gel properties favorable for intra-articular delivery, including reduced swelling and degradation and prolonged drug release, while simultaneously promoting M1-to-M2 macrophage polarization as an immunomodulatory agent. The resulting oxMCP/NOCC/BBR hydrogel promoted M1-to-M2 macrophage polarization, protected chondrocytes from apoptosis and ECM degradation, and alleviated osteoarthritis progression.

These strategies were based on the hypothesis that oxMCP, acting as both a Gal-3-binding backbone and a crosslinkable matrix, would form an injectable hydrogel further empowered by BBR to provide network integrity and therapeutic bioactivity for OA treatment (Fig. 1). Notably, no study to date has reported the development of an MCP-based injectable hydrogels, highlighting a critical gap in translating the MCP's bioactivity into practical delivery platforms. In this study, we developed an injectable hydrogel based on oxMCP, which served as both a Gal-3-sequestering therapeutic polysaccharide and a chemically crosslinkable scaffold. The physicochemical properties, injectability, release kinetics, and therapeutic efficacy *in vitro* and in a surgically induced OA model of the resulting oxMCP/NOCC/BBR hydrogel were systematically evaluated. By integrating Gal-3 sequestration and dynamic crosslinking within a single macromolecular framework, this platform presents a rationally designed hydrogel system for localized and sustained OA treatment.

## 2 Materials and methods

### 2.1 Materials

CP (P9135, galacturonic acid ≥74.0 %, Mw 485 kDa, degree of esterification (D.E.) 57.86 %) [34], BBR, lipopolysaccharide (LPS), IL-1β, phorbol 12-myristate 13-acetate (PMA), Cell Counting Kit-8 (CCK-8), 2′,7′-dichlorodihydrofluorescein diacetate (DCFH-DA), 1-ethyl-3-(3-dimethylaminopropyl) carbodiimide (EDC), N-hydroxysuccinimide (NHS), fluorescein amine, and bovine serum albumin (BSA) were purchased from Sigma-Aldrich (St. Louis, MO, USA). NOCC was synthesized according to our previous studies and characterized to have a molecular weight of 230 kDa, an O-substitution degree of 0.36, and an N-substitution degree of 0.45 [35]. HA (Mw 15–30 kDa) was purchased from NOVA Pharma & Liposome Biotech (Kaohsiung, Taiwan). Interferon-γ (IFN-γ), interleukin-4 (IL-4), and interleukin-13 (IL-13) were purchased from ProSpec-Tany TechnoGene (Rehovot, Israel). Fetal bovine serum (FBS) was obtained from Thermo Fisher Scientific (Waltham, MA, USA). Dulbecco's modified Eagle medium (DMEM), RPMI-1640 medium, penicillin, and streptomycin sulfate were purchased from Gibco (Rockville, MD, USA). The human chondrocyte cell line C28/I2 was obtained from Merck (SCC043, Darmstadt, Germany), the human monocytic cell line THP-1 was purchased from ATCC (TIB-202, Manassas, VA, USA), and the mouse fibroblast cell line L929 was obtained from the Food Industry Research and Development Institute (FIRDI, Hsinchu, Taiwan).

### 2.2 Preparation of MCP

CP (7.5 g) was dissolved in 500 mL of ultrapure water and stirred using a magnetic stirrer until a clear solution was obtained. The pH was adjusted to 10 by the dropwise addition of 10 M NaOH, which yielded a clear brown solution. The mixture was then stirred at 55 °C for 1 h and subsequently cooled to room temperature. Next, the pH was adjusted to 3 by the dropwise addition of 3 M HCl, and the mixture was stirred at room temperature for 24 h, during which a turbid, milky-white suspension gradually formed. The pH was next adjusted to 6.8 using 3 M NaOH. MCP was precipitated by the addition of 95 % ethanol in a 1:4 (v/v) ratio and allowed to stand for 10 min. The resulting precipitate was collected by centrifugation at 10,000 rpm and 20 °C for 10 min. Acetone was added to the precipitate at the same volume ratio, followed by mechanical disruption and another quiescent period for 10 min. After a second centrifugation under the same conditions, the final MCP product was dried at 37 °C and stored at 4 °C. The D.E. was determined by ^1^H nuclear magnetic resonance (NMR) spectroscopy using an Agilent 600 MHz spectrometer (Santa Clara, CA, USA). MCP was dissolved in D_2_O, and D.E. was calculated by integrating the methyl ester proton signal (∼4.3 ppm) relative to the anomeric proton signals of galacturonic acid residues (∼4.9–5.2 ppm).

### 2.3 Preparation of oxMCP

MCP (0.5 g) was dissolved in 75 mL of ultrapure water under magnetic stirring. Separately, 25 mL of an NaIO_4_ solution was prepared at various molar ratios relative to the repeating units of MCP, and subsequently added to the MCP solution. The reaction mixture was stirred at room temperature in the dark for 24 h. Upon completion, 95 % ethanol was added at a 1:4 (v/v) ratio to precipitate the oxidized product. After allowing it to stand for 10 min, the precipitate was collected by centrifugation at 10,000 rpm and 20 °C for 10 min. Acetone was then added at the same volume ratio (1:4), and the sample was mechanically disrupted and left to stand for another 10 min. A second centrifugation under the same conditions was performed to collect the final product. The obtained oxMCP was dried at 37 °C and stored at 4 °C. The degree of oxidation was determined by titration with hydroxylamine hydrochloride.

### 2.4 Preparation of hydrogels

Conventional hydrogel fabrication methods typically involve physical blending or chemical crosslinking of natural or synthetic polymer solutions [36]. A 6 % (w/w) solution of oxMCP (oxidized using NaIO_4_ at 0.6 g/g MCP) was prepared in phosphate-buffered saline (PBS). Separately, NOCC solutions at concentrations ranging from 1 % to 6 % (w/w) were prepared in PBS and stirred with a magnetic stirrer until fully dissolved. Each NOCC solution was then mixed with the 6 % oxMCP solution at a 1:1 (v/v) ratio. The resulting mixtures were incubated at 37 °C, and the gelation behavior was monitored to determine gelation rates at different NOCC concentrations. For oxMCP/NOCC/BBR hydrogels, BBR was first dissolved in a 6 % NOCC solution at final concentrations ranging 0.5–2.0 mg/mL. Each BBR-containing NOCC solution was then mixed with the 6 % oxMCP solution at a 1:1 (v/v) ratio, and the mixtures were incubated at 37 °C for gelation.

### 2.5 Characterization of the hydrogels

Gel formation was initially assessed by an inverted tube test at 37 °C, and successful gelation was defined as the point at which the hydrogel no longer flowed upon inversion of the vial. The rheological properties of the hydrogels were evaluated using a rheometer (MCR 302, Anton Paar, Graz, Austria). A sample volume of 0.5 mL was loaded for each measurement. Time-sweep (1 Hz) and frequency-sweep (1–100 rad/s) tests were conducted using the instrument's built-in protocol. Self-healing properties were assessed by strain-sweep measurements, where the applied shear strain was increased from 1 % to 500 %. The critical strain was defined as the intersection point of the storage modulus (G′) and loss modulus (G″). To further evaluate their self-healing behavior, hydrogels were subjected to cyclic oscillatory strain tests alternating between low and high strain amplitudes.

The surface morphology and pore size distribution of freeze-dried hydrogels were examined using scanning electron microscopy (SEM; SU3500, Hitachi, Tokyo, Japan), and pore diameters were quantified using ImageJ software (National Institutes of Health, Bethesda, MD, USA). The mechanical properties were determined using a Texture Profile Analyzer (TA-XT2, Stable Micro Systems, Haslemere, UK) at 37 °C and 70 % relative humidity. Hydrogel samples (10 × 10 × 4 mm, W × L × H) were subjected to uniaxial compression to 90 % of their original height at a rate of 0.1 mm/s.

The swelling behavior and degradation rate were also evaluated. After complete gelation, the hydrogels were immersed in 10 vol of RPMI-1640 medium and incubated at 37 °C. At each predetermined time point, samples were gently blotted to remove surface water and weighed. The swelling ratio was calculated using Eq. (1):

(1) $$\text{Swelling}\;\text{ratio}\;\left(\%\right)=\left(\frac{W_{s}}{W_{i}}\right)\times 100\;\text{;}$$

where *Wi* is the initial dry weight and *Ws* is the swollen weight at the given time point.

To assess degradation, swollen samples were freeze-dried and weighed. The degradation rate was calculated as the percentage of remaining dry weight using Eq. (2):

(2) $$\text{Remaining}\;\text{weight}\;\left(\%\right)=\left(\frac{W_{t}}{W_{i}}\right)\times 100\;\text{;}$$

where *Wt* is the dry weight of the sample at the selected time point.

### 2.6 Spectroscopic and structural analyses

The chemical structures of the samples were characterized by ^1^H NMR spectroscopy using an Agilent DD2 600 MHz NMR spectrometer and Fourier transform infrared (FT-IR) spectroscopy (FTS-155, Bio-Rad Laboratories, Boston, MA, USA). The molecular weight of MCP was determined by gel permeation chromatography (GPC) on a Shimadzu LC-20 Prominence system equipped with a refractive index detector and a TSKgel G4000PWxL column (Tokyo, Japan). Ultrapure water was used as the mobile phase at a flow rate of 0.5 mL/min. Filtered MCP samples (10 mg/mL; injection volume: 5 μL) were analyzed, and the molecular weight was calibrated using a dextran standard kit (Agilent Technologies, CA, USA). Crystalline structures of the samples were examined using an x-ray diffractometer (D2 Phaser, Bruker, Billerica, MA, USA) equipped with a Ni filter and Cu Kα radiation (λ = 0.154 nm), operated at 15 kV and 20 mA. Data were collected in continuous scan mode at a scanning rate of 5.3°/min over a 2θ range of 5°–80°.

### 2.7 BBR release

The oxMCP/NOCC/BBR hydrogels were immersed in PBS (pH 7.4) at a hydrogel-to-medium volume ratio of 2:8. At predetermined time points, 200 μL of the release medium was withdrawn for analysis, and the remaining medium was completely replaced with fresh PBS to maintain sink conditions. The concentration of BBR in each collected sample was determined by measuring the absorbance at 343 nm on a microplate spectrophotometer (Spark, Tecan, Switzerland). A standard calibration curve was established using known BBR concentrations to convert absorbance values to concentrations. The cumulative release percentage was calculated using the following equation:

(3) $$\text{Cumulative}\;\text{release}\;\left(\%\right)=\left(\frac{\sum_{i=1}^{t}C_{i}\times V}{M_{0}}\right)\times 100\text{;}$$

where *t* is the total number of sampling time points considered, *C*_*i*_ is the concentration of BBR in the release medium at time point *i*, *V* is the volume of release medium collected at each time point (200 μL), and *M*_*0*_ is the total amount of BBR initially loaded in the hydrogel.

### 2.8 Visualization and Quantification of Gal-3 binding in hydrogels

To visualize the binding and colocalization of Gal-3 with oxMCP-based hydrogel, a dual-labeling fluorescence approach was employed. oxMCP was fluorescently labeled with Cy7 amine. Briefly, 0.5 g of oxMCP was dissolved in 100 mL of 50 mM MES buffer (pH 6.5), followed by the dropwise addition of 6.25 mg Cy7 amine (dissolved in 250 μL DMSO). Sodium cyanoborohydride (NaBH_3_CN, 6.3 mg) was then added to stabilize the Schiff base linkages. The reaction was performed at 4 °C for 16 h under light-protected conditions. To remove unreacted Cy7, the product was precipitated by cold acetone (1:5, v/v), collected by centrifugation, and washed with acetone twice. The purified Cy7-labeled oxMCP was dried and stored at −20 °C in the dark.

Recombinant human Gal-3 (ProSpec, Rehovot, Israel; CYT-606) and bovine serum albumin (BSA, Sigma-Aldrich, St. Louis, USA) were fluorescently labeled with rhodamine isothiocyanate (RITC, Chem-Impex, Wood Dale, USA). For Gal-3, 50 μg protein was incubated with 2.6 μg RITC (molar ratio ∼2.5:1) in 100 μL of 0.1 M sodium bicarbonate buffer (pH 9.0) for 2h at room temperature in the dark. For BSA, 50 μg protein was labeled with 2.44 μg RITC (molar ratio ∼6:1) under identical conditions to normalize the fluorescence intensity per unit protein mass. Free RITC was removed by ultrafiltration using 3 kDa molecular weight cut-off centrifuge tubes (Vivaspin 2®, Sartorius, USA).

Hydrogels were formed by mixing Cy7-labeled oxMCP, NOCC, and BBR as described in section 2.4, with 50 μL of pre-gel solution cast onto 20 mm glass coverslips for *in situ* gelation. After curing, the hydrogel-coated coverslips were incubated with 200 μL of RITC-labeled Gal-3 or BSA (25 μg/mL in PBS) at 37 °C for 2 h to allow protein diffusion and binding. Unbound proteins were removed by PBS washing (3 × , 20 min each). Confocal imaging was performed using a Leica Stellaris 8 system (Leica Microsystems, Wetzlar, Germany) to visualize the spatial distribution and overlap of Cy7-labeled oxMCP and RITC-labeled proteins. BSA-RITC served as a nonspecific adsorption control.

In parallel, Gal-3 adsorption was quantified by measuring the decrease in supernatant fluorescence intensity. After incubation with Gal-3-RITC, supernatants were collected and analyzed using a microplate reader (Ex/Em: 530/590 nm). BSA-RITC was included as a nonspecific control, and samples without hydrogels served as blanks. Adsorption efficiency percentage was calculated using the following equation:

(4) $$\text{Adsorption}\;\text{efficiency}\;\left(\%\right)=\left(\frac{F_{0}-{\;F}_{t}}{F_{0}}\right)\times 100\text{;}$$

where *F*_*0*_ and *F*_*t*_ represent the fluorescence intensity before and after incubation, respectively.

### 2.9 Cell culture and cell viability assay

L929 fibroblasts and C28/I2 chondrocytes were cultured in DMEM, while THP-1 monocytes were maintained in RPMI-1640, supplemented with 10 % FBS and 1 % penicillin–streptomycin. Cells were seeded in 24-well plates and incubated with preformed hydrogels for 24 h at 37 °C in a 5 % CO_2_ atmosphere. Cell viability was assessed using the CCK-8 assay, followed by measurement of absorbance at 450 nm using on a microplate reader (Spark).

### 2.10 Immunomodulatory capacity

THP-1 monocytes were seeded at a density of 1 × 10^6^ cells per well in 6-well plates and treated with 100 nM PMA for 24 h to induce differentiation into M0 macrophages. To further polarize cells, M1 macrophages were induced with 100 ng/mL LPS and 20 ng/mL IFN-γ, while M2 macrophages were induced with 25 ng/mL IL-4 and 25 ng/mL IL-13 for 72 h with daily medium refreshment [37]. To assess macrophage polarization, cells were stained with anti-CD38-FITC (clone HIT2) and anti-CD206-APC (clone 15-2) (Sigma-Aldrich) mouse monoclonal antibodies, followed by a flow cytometric analysis using the Attune® NxT acoustic focusing cytometer (Invitrogen, Carlsbad, CA, USA). In functional assays, M0 macrophages were co-treated with MCP, oxMCP, or NOCC (each at 600 μg/mL) and LPS (100 ng/mL)/IFN-γ (20 ng/mL) for 72 h to assess their anti-inflammatory effects. In parallel, oxMCP/NOCC and oxMCP/NOCC/BBR hydrogels were applied at equivalent concentrations under the same conditions, with BBR present at 100 μg/mL. Supernatants were collected, and inflammatory cytokines, including TNF-α, IL-1β, IL-4, IL-6, and IL-10, were analyzed using enzyme-linked immunosorbent assay (ELISA) kits (Sigma-Aldrich).

### 2.11 Gal-3-binding affinity

To evaluate the Gal-3-binding affinity, MCP and oxMCP were labeled with a fluorescent amine. Briefly, MCP (1 g) was dissolved in 250 mL of ultrapure water containing 300 mg of EDC, 450 mg of NHS, and 0.9 g of MES. The mixture was stirred at 40 °C for 45 min to activate carboxyl groups. Unreacted EDC and NHS were removed by precipitation with seven volumes of ethanol. Activated MCP was redissolved in 20 mL of PBS and reacted with 80 mg of fluorescent amine (25 mg/mL in DMSO) at room temperature for 48 h. Labeled products were precipitated with cold methanol (−20 °C), filtered under a vacuum, washed twice with methanol, and dried for storage at −20 °C, yielding MCP-FL and oxMCP-FL. LPS (100 ng/mL) and IFN-γ (20 ng/mL) were used to induce THP-1 cells (3 × 10^5^ cells/dish, seeded in 35-mm glass-bottom dishes; Alpha Plus, Taiwan) into M1 macrophages for 24 h. These cells served as a Gal-3 overexpression model for a binding analysis. After induction, cells were fixed with 4 % paraformaldehyde and incubated with MCP-FL or oxMCP-FL under identical conditions. Unbound polymers were removed by PBS washing. Gal-3 expression was confirmed by immunostaining with a primary antibody (GTX125897, GeneTex, Cambridge, MA, USA). The binding affinity was visualized using a Leica Stellaris 8 confocal laser scanning microscope (Leica Microsystems, Wetzlar, Germany) and quantified by measuring the fluorescence intensity with ImageJ software.

### 2.12 Chondroprotective effect

C28/I2 chondrocytes were seeded at 2 × 10^5^ cells/well in 6-well plates and stimulated with IL-1β (10 ng/mL) for 48 h to mimic an inflammatory environment. MCP and oxMCP (600 μg/mL) were co-incubated with IL-1β-treated cells for another 48 h. In parallel, oxMCP/NOCC and oxMCP/NOCC/BBR hydrogels were applied under the same conditions at equivalent polymer concentrations, with BBR present at 100 μg/mL. Apoptosis was evaluated using an Annexin V-FITC/PI double staining kit (Sigma-Aldrich), followed by a cytometric analysis with an Attune® NxT acoustic focusing cytometer (Invitrogen). Expression of matrix metalloproteinase-13 (MMP-13) was assessed by immunofluorescence (IF) using an anti-MMP13 antibody (AB39012, Abcam, Branford, CT, USA). Intracellular reactive oxygen species (ROS) levels were detected using a DCFH-DA assay. Briefly, cells were incubated with 10 μM DCFH-DA in serum-free medium for 30 min at 37 °C in the dark, followed by washing with PBS to remove any excess probe. MMP-13 expression and ROS levels were visualized using confocal laser scanning microscopy (Leica Stellaris 8, Leica Microsystems) and quantified by measuring the fluorescence intensity with ImageJ software.

### 2.13 Hulth model in SD rats

All experimental designs and procedures were approved by the Institutional Animal Care and Use Committee (IACUC) of Taipei Medical University (LAC2024-0229). Animals were housed and cared for in accordance with ARRIVE guidelines at an accredited facility. An OA model was established in female Sprague-Dawley (SD) rats (8 weeks old, n = 25) using the Hulth surgical method. All rats underwent bilateral knee surgery under general anesthesia with inhaled isoflurane (induction at 3 %, maintenance at 1.5 %). The procedure involved transection of the anterior cruciate ligament (ACL) and medial collateral ligament (MCL), and removal of the medial meniscus. Joint instability was confirmed using an anterior drawer test. Postoperative analgesia was provided by subcutaneous injection of meloxicam (1 mg/kg body weight) once daily for 3 days. In addition, enrofloxacin (18 mg/300 mL) was administered in drinking water for 3 days (estimated intake: 50 mL/day) for infection prophylaxis.

### 2.14 Intra-articular treatment and evaluation

Intra-articular injections were initiated 2 weeks after surgery. To enhance joint retention and reduce the diffusion of MCP, a 1 % HA solution was used as the carrier. MCP or oxMCP (3 %) was mixed with 1 % HA and administered at a dose of 10 μL per knee every 2 weeks, for a total of three injections. The hydrogel groups received treatments at the same frequency and injection volume. To monitor joint inflammation, the transverse diameter of the knee joint was measured on days 0, 7, 14, 21, and 28 using digital calipers. Swelling was quantified as the change in joint diameter relative to the baseline, calculated using the following equation:

(5) $$\Delta D=\left(D_{x}-D_{0}\right)\text{;}$$

where Δ*D* is the degree of swelling, *D*_*x*_ is the transverse diameter of the knee joint at each time point (day 7, 14, 21, or 28), and *D*_*0*_ is the baseline diameter measured on day 0.

Synovial fluid was collected at week 4 using a lavage technique. Briefly, 10 μL of sterile PBS was injected into the joint cavity with a 30 G needle, followed by gentle massage. The lavage fluid was then aspirated and stored for further ELISA analyses. Cytokines including TNF-α, IL-1β, IL-4, IL-6, and IL-10 were quantified using commercial ELISA kits (Sigma-Aldrich) according to the manufacturers’ protocols. At week 8, rats were sacrificed, and the entire knee joint (including the distal femur and proximal tibia) was harvested and fixed in 4 % paraformaldehyde, decalcified with 10 % EDTA, embedded in paraffin, and sectioned at 4 μm thickness for a subsequent histological analysis.

### 2.15 Histological staining

Hematoxylin and eosin (H&E) staining was used to evaluate the overall tissue morphology. Sections were stained with Mayer's hematoxylin (TA01MH, Biotna Scientific, Fremont, CA, USA) for 3 min, rinsed, counterstained with eosin Y solution (TA01ES, Biotna Scientific, Fremont, CA, USA) for 1 min, dehydrated, cleared, and mounted. Masson's trichrome staining was performed to visualize collagen fibers. Sections were stained with Weigert's iron hematoxylin, Biebrich scarlet-acid fuchsin, and aniline blue using a commercial staining kit (TASS01, Biotna Scientific, Fremont, CA, USA), with differentiation steps in phosphomolybdic-phosphotungstic acid and 1 % acetic acid, followed by dehydration and mounting. Safranin O-Fast Green staining was used to assess cartilage matrix proteoglycans. Sections were stained with Weigert's iron hematoxylin, Fast Green FCF, and safranin O using a dedicated kit (TASS10, Biotna Scientific, Fremont, CA, USA), with a brief 1 % acetic acid rinse, dehydration, and mounting. Images were acquired using Motic Easyscan Pro 6 (Motic, Hong Kong, China).

### 2.16 Statistical analysis

All data are presented as the mean ± standard deviation (SD), and statistical analyses were performed using SPSS Statistics 19 software (IBM, NY, USA). Comparisons between multiple groups were conducted using a one-way analysis of variance (ANOVA), followed by Tukey's post-hoc test to determine group differences. A *p* value of <0.05 was considered statistically significant. In figures, different lowercase letters (e.g., a, b, c, d) indicate statistically significant differences between groups (*p* < 0.05).

## 3 Results and discussion

### 3.1 Structural transformation and physicochemical characterization of oxMCP

CP is a plant-derived polysaccharide primarily composed of α-(1 → 4)-linked D-galacturonic acid units, in which the majority of carboxyl groups are methyl-esterified. Due to its high molecular weight and D.E., native CP exhibits poor water solubility, which limits its applicability in aqueous biomedical systems such as injectable hydrogels [38]. To improve its solubility and bioactivity, CP was converted into MCP via a sequential pH-controlled treatment. Under alkaline conditions (pH 10.0), β-elimination reactions cleaved the homogalacturonan (HG) backbone and partially removed methyl ester groups, resulting in a significant reduction in molecular weight and an increase in the carboxylate content [39]. Subsequent acid treatment (pH 3.0) induced partial hydrolysis of neutral sugar side chains, especially arabinan and galactan moieties from rhamnogalacturonan-I (RG-I) regions [16,39]. This hydrolytic trimming facilitated the exposure of previously masked β-(1 → 4)-galactan epitopes, which are recognized as key Gal-3-binding motifs [16]. The resulting MCP is a water-soluble, low-methoxyl polysaccharide (typically <30 kDa), consisting of low-molecular-weight fragments derived from both the HG and RG-I domains, and which retains structural features relevant to Gal-3 binding and immunomodulation [16,39,40].

**Fig. 2:**
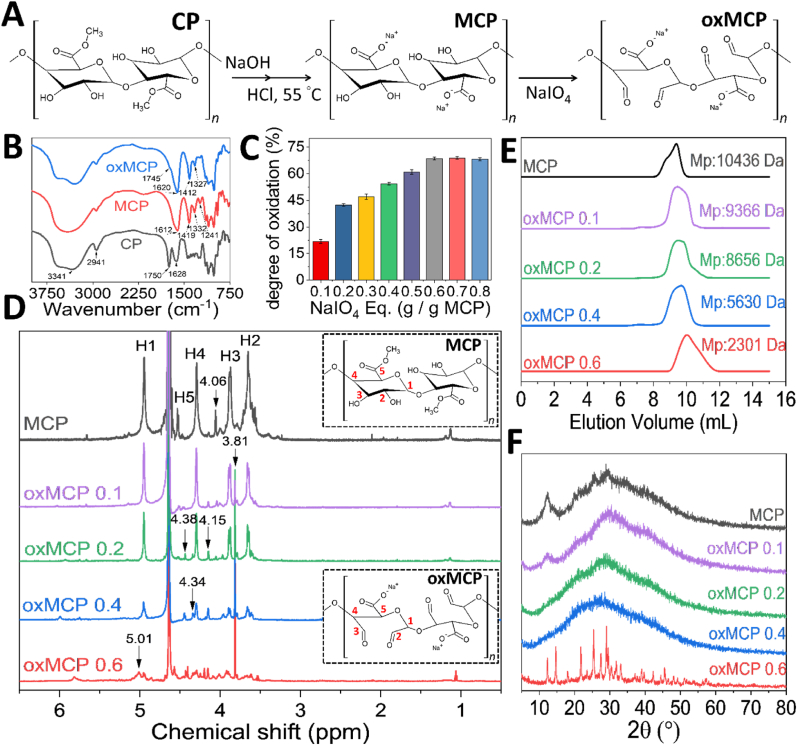
Structural and physicochemical characterization of MCP and oxMCP. (A) Schematic illustration of periodate oxidation of MCP, using homogalacturonan (HG) residues as representative units to show dialdehyde formation for Schiff base crosslinking. (B) FTIR spectra. (C) Degree of oxidation determined by hydroxylamine hydrochloride titration. (D) ^1^H NMR spectra. (E) GPC analysis. (F) XRD patterns.

To expand the biomedical applicability of MCP, we introduced a functional oxidation step using NaIO_4_ to generate dialdehyde-functionalized MCP (oxMCP) suitable for dynamic covalent hydrogel formation. Subsequent oxidation with NaIO_4_ selectively cleaved the C2–C3 vicinal diol bonds in the galacturonic acid residues, producing dialdehyde groups [18]. The resulting product, oxMCP, possessed reactive aldehyde moieties capable of forming covalent imine (Schiff base) bonds with amines, which is critical for hydrogel crosslinking (Fig. 2A). In this design, oxMCP served both as a crosslinkable matrix component and a bioactive polysaccharide. The resulting dynamic network can be administered via injection, offering enhanced local retention and sustained release at lesion sites. Moreover, the reversible nature of Schiff base bonds may endow the hydrogel with a self-healing ability, which is especially beneficial for maintaining material integrity under joint-associated mechanical stresses [21].

FTIR spectroscopy was used to monitor the structural evolution from CP to MCP and oxMCP (Fig. 2B). CP exhibited a broad band at 3341 cm^−1^ corresponding to O–H stretching, and a peak at 2941 cm^−1^ associated with C–H stretching of methyl esters. The strong absorbance at 1750 cm^−1^ was assigned to ester carbonyl stretching (–COOCH_3_), while the band at 1628 cm^−1^ was attributed to asymmetric stretching of carboxylate groups (–COO^-^). In MCP, the disappearance of the 1750 cm^−1^ peak confirmed de-esterification, accompanied by intensified bands at 1612 and 1419 cm^−1^, corresponding to asymmetric and symmetric –COO^-^ stretching [16]. New peaks at 1332 and 1241 cm^−1^, assigned to C–OH bending and C–O stretching, suggest localized structural reorganization within the polysaccharide backbone or side-chain regions. Following periodate oxidation, oxMCP showed a new peak at 1745 cm^−1^, indicative of aldehyde C=O stretching and confirming successful formation of dialdehyde groups. Shifts in carboxylate-associated bands (1620 and 1412 cm^−1^) and changes near 1327 cm^−1^ are likely due to ring-opening oxidation of galacturonic acid residues, which perturbed the electronic environment and vibrational behavior of adjacent –COO^-^ and –OH groups. These spectral features collectively validated the stepwise conversion of CP to oxMCP and the introduction of functional aldehyde groups for Schiff base-mediated hydrogel crosslinking.

To determine the optimal degree of aldehyde functionalization and to better understand the relationship between oxidative ring opening and the physicochemical properties of MCP, a series of oxMCP samples were prepared using various amounts of NaIO_4_. The degree of oxidation was quantified by hydroxylamine hydrochloride titration, which specifically detects aldehyde groups. As shown in Fig. 2C, the oxidation degree increased with the NaIO_4_-to-MCP ratio. At 0.1 g of NaIO_4_ per gram of MCP, the oxidation degree was 21.8 %, and increased progressively to 42.4 %, 47.0 %, 54.4 %, 61.0 %, and 68.6 % at 0.2–0.6 g/g MCP, respectively. Beyond this point, the oxidation efficiency plateaued, reaching 68.9 % and 68.2 % at 0.7 and 0.8 g/g MCP. These results indicated that periodate-mediated oxidation of MCP achieved a practical maximum aldehyde content of approximately 70 % when using 0.6 g of NaIO_4_ per gram of MCP, beyond which additional oxidant did not further enhance oxidation. This saturation was likely due to the limited accessibility of residual vicinal diol groups and the onset of non-specific oxidative pathways. In particular, excess periodate may have given rise to reactive oxidative species or byproducts, which can contribute to glycosidic bond cleavage and partial depolymerization of the polysaccharide backbone [41,42]. Based on these findings, oxMCP with an oxidation degree of ∼70 % was selected for subsequent experiments to maximize the crosslinking potential while preserving the molecular integrity.

To further investigate the structural consequences of periodate oxidation, we employed ^1^H NMR and gel permeation chromatography (GPC) to respectively examine chemical transformations at the molecular level and changes in macromolecular size. In Fig. 2D, ^1^H NMR spectra of MCP and oxMCP with varying oxidation degrees were obtained in D_2_O (Fig. 2D). The spectrum of MCP exhibited well-resolved signals corresponding to protons on galacturonic acid residues, including H1 (*δ* ∼4.9 ppm), H2 (*δ* ∼3.6 ppm), H3 (*δ* ∼3.8 ppm), H4 (*δ* ∼4.3 ppm), and H5 (*δ* ∼4.5 ppm), consistent with intact pyranose ring structures [43]. As the oxidation degree increased, these backbone proton signals exhibited a progressive reduction across oxMCP 0.1 to 0.6, reflecting oxidative ring opening of the galacturonic acid units. A distinct peak at *δ* 4.06 ppm was observed with MCP, which rapidly shifted to *δ* 4.15 following periodate oxidation. This signal was likely attributable to H4 protons of neutral sugar residues, such as arabinose, xylose, or glucose, originating from residual side chains in RG-I regions that contain vicinal diols susceptible to selective oxidative cleavage [16,24]. Concurrently, new signals emerged at *δ* 5.01, 4.38, 4.34, and 3.81 ppm in the oxidized samples, with their intensities increasing proportionally to the degree of oxidation. The peak at *δ* 5.01 ppm, most distinguishable in the highly oxidized sample (oxMCP 0.6), was assigned to the anomeric proton (C1–H) of newly formed reducing ends, providing direct evidence of periodate-induced chain scission. The signal at *δ* 4.38 ppm is assignable to protons at the C5 position, which may have experienced mild deshielding due to structural perturbations induced by oxidation at neighboring residues. The signal at *δ* 4.34 ppm may be attributed to protons at the C4 position, deshielded by adjacent electron-withdrawing groups such as a newly formed aldehyde at C3 and a native carboxylic acid at C6. In contrast, the signal at *δ* 3.81 ppm likely arose from the –CH proton in aldehyde hydrate (–CH(OH)_2_) structures generated at vicinal diol cleavage sites, such as C2 and C3 [44]. At the highest oxidation level (oxMCP 0.6), a broad range of additional peaks appeared throughout the 3.5–5.5 ppm region, likely reflecting a heterogeneous mixture of oligosaccharide products generated by oxidative cleavage. In addition to qualitative spectral changes, the D.E. was calculated based on the relative integration of H1 and H5 signals [45]. The DE of commercial CP was 57.86 % [34], while that of MCP was reduced to 7.36 %, confirming efficient demethylation during alkaline pretreatment. Collectively, these spectral changes provide molecular-level evidence for both demethylation and ring-opening oxidation of MCP, confirming the successful introduction of reactive aldehyde functionalities and suggesting the presence of partial scission and oligosaccharide formation at higher oxidation levels.

Following the NMR analysis, GPC was used to assess the molecular weight distribution and further probe potential chain fragmentation. In Fig. 2E, the original MCP showed a unimodal peak with a peak molecular weight (Mp) of 10,436 Da and a number-average molecular weight (Mn) of 12,120 Da. Upon oxidation, a progressive decrease in molecular weight was observed with increasing NaIO_4_ equivalents. For oxMCP 0.1 and 0.2, Mp values decreased modestly to 9366 and 8656 Da, respectively, while Mn remained relatively stable, suggesting that minor chain scission occurred without significant fragmentation. However, at higher oxidation levels (oxMCP 0.4 and 0.6), molecular weights markedly dropped (Mp = 5630 and 2301 Da; Mn = 5781 and 3270 Da), confirming that periodate-induced cleavage became increasingly effective. This trend was also reflected in the reduction of the weight-average molecular weight (Mw) and the narrowing of the polymer size, while the polydispersity index (PDI) initially increased (from 1.63 to 2.29 at oxMCP 0.1) before decreasing at oxMCP 0.6 (PDI = 1.56), indicating that higher degrees of oxidation not only induced chain cleavage but also removed high-molecular-weight fractions, yielding a more-homogeneous oligomeric population. These GPC results are consistent with the NMR-observed spectral broadening and support the hypothesis of partial chain scission and oligosaccharide formation at higher oxidation degrees.

To evaluate changes in molecular packing and crystallinity during oxidation, an x-ray diffraction (XRD) analysis was performed on MCP and oxMCP samples. In Fig. 2F, native MCP exhibited multiple characteristic reflections, including a crystalline peak at 2θ = 12.3°, which was attributed to short-range ordering of homogalacturonan (HG) segments within the semicrystalline domains, owing to their linear, hydrogen bond-rich structure [46]. Additional weaker peaks were observed at 2θ = 19.9°, 25.4°, and 34.4°, superimposed on a broad amorphous halo centered around 29.6°, reflecting partial ordering among galacturonic acid (GalA) segments through interchain hydrogen bonding. Upon periodate oxidation, the 12.3° peak progressively decreased in intensity and had entirely disappeared by oxMCP 0.2, indicating disruption of these ordered HG domains due to cleavage of vicinal diols and loss of hydrogen-bonding interactions. Meanwhile, the broad amorphous halo gradually shifted toward lower angles, reaching 2θ = 27.3° in oxMCP 0.4, suggesting a gradual increase in intermolecular spacing and reduced packing efficiency, likely caused by ring opening and enhanced molecular flexibility. These changes collectively indicated a transition from semi-crystalline to more-amorphous organization as oxidation proceeded. Notably, oxMCP 0.6 displayed a distinct set of sharp, narrow peaks across the 2θ range, in contrast to the amorphous patterns observed in less-oxidized samples. These features are consistent with the formation of short-chain oligosaccharides containing only a few monosaccharide units, which can adopt more-regular conformations and form crystalline domains upon solidification. The emergence of multiple diffraction signals further supported the presence of oligosaccharide products with various chain lengths, resulting in diverse packing behaviors and corroborating the GPC and 10.13039/501100004182NMR evidence of progressive oxidative fragmentation. Collectively, these findings demonstrate that periodate oxidation not only introduced aldehyde functionalities for hydrogel formation, but also modulated the molecular conformation and composition of MCP in ways that may have increased the accessibility of Gal-3-binding motifs. These structural transformations are anticipated to enhance the biological activity of oxMCP and support its further development as an immunomodulatory and hydrogel-forming biomaterial.

### 3.2 Structural transformation enables Gal-3 binding and biofunctional improvements in oxMCP

**Fig. 3:**
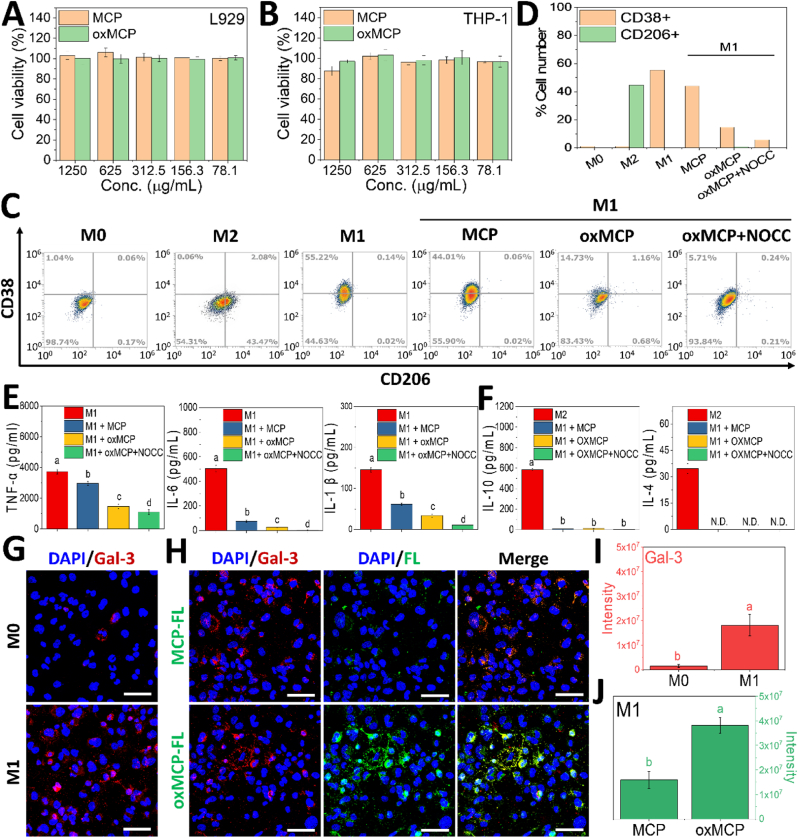
Anti-inflammatory activity and the Gal-3-binding affinity of oxMCP. (A–B) Cell viability of L929 and THP-1 cells (*n* = 6). (C–D) Flow cytometric analysis of CD38^+^ M1 and CD206^+^ M2 macrophage populations (*n* = 3). (E–F) Cytokine secretion profiles of M1 markers (TNF-α, IL-6, and IL-1β) and M2 markers (IL-10 and IL-4) (*n* = 6). (G) Confocal images showing Gal-3 expression in M0 and M1 macrophages. (H) Confocal colocalization analysis of fluorescently labeled MCP-FL and oxMCP-FL (green) with Gal-3 (red) in M1 macrophages, reflecting their relative binding affinity and spatial overlap. (I, J) Quantification of Gal-3 (red) and MCP/oxMCP (green) fluorescence intensities in (G) and (H), respectively, using ImageJ software (*n* = 3). Scale bars: 50 μm. Data are presented as the mean ± standard deviation; *p* < 0.05.

Building on the pronounced structural and physicochemical alterations of oxMCP, we next evaluated its biological activity as it relates to OA treatment. Given the critical role of inflammation in OA progression, we first examined the immunomodulatory effects of oxMCP. The cytocompatibility of oxMCP was confirmed with both L929 fibroblasts and THP-1 monocytes, with cell viability remaining above 90 % across all tested concentrations (Fig. 3A and B), comparable to MCP. Treatment with oxMCP markedly reduced the proportion of CD38^+^ M1-type macrophages (Fig. 3C and D), exhibiting a 3.35-fold stronger inhibitory effect compared to MCP and highlighting its enhanced anti-inflammatory capacity. As NOCC was intended to be combined with oxMCP for hydrogel fabrication, its immunomodulatory contribution was also assessed *in vitro*. Interestingly, co-treatment with NOCC further decreased the M1 population, likely due to its intrinsic anti-inflammatory properties [47]. However, no significant increase in CD206^+^ M2-like macrophages was observed, suggesting that oxMCP primarily functions by suppressing pro-inflammatory activation rather than promoting M2 polarization. This was further supported by cytokine profiling, in which oxMCP effectively downregulated the M1-associated markers, TNF-α, IL-6, and IL-1β (Fig. 3E), while failing to induce M2-related cytokines, such as IL-10 and IL-4 (Fig. 3F). These findings highlight the superior immunomodulatory activity of oxMCP relative to MCP, especially in attenuating M1-type macrophage activation.

Given the well-established role of Gal-3, a β-galactoside-binding lectin, in promoting macrophage activation and cartilage degeneration [13,14], we considered that the enhanced anti-inflammatory activity of oxMCP may have resulted from an increased Gal-3-binding affinity conferred by its structural modifications. In light of the structural transformations observed in Section 3.1, the enhanced Gal-3-binding activity of oxMCP may have arisen from multiple structural effects induced by periodate oxidation. First, oxidative ring opening disrupted the rigid pyranose backbone of HG residues, increasing polymer flexibility, reducing steric hindrance, and facilitating access to the CRD of Gal-3 [19]. Second, the reduction in molecular weight and the removal of non-functional side chains may have enhanced the relative exposure and accessibility of bioactive domains [25], such as Gal-3-binding epitopes. Finally, cleavage of branched neutral sugars, such as α-arabinofuranose units in AG-II structures of the RG-I domain [16], may have shifted the local conformation toward a more-linear AG-I-like configuration, which is known to exhibit stronger Gal-3 affinity [26]. Taken together, these structural features may have synergistically enhanced the Gal-3-binding capability of oxMCP.

To assess the enhanced Gal-3-binding capacity of oxMCP, THP-1 monocytes polarized to the M1 phenotype, a condition known to upregulate Gal-3 expression, were employed (Fig. 3G–I). This LPS/IFN-γ-induced M1 model reflects the inflammatory environment of osteoarthritic joints, in which Gal-3 is not only highly expressed but also functions as a pro-inflammatory amplifier that enhances TLR4/NF-κB signaling [28,48,49]. Therefore, it provides a physiologically relevant platform for evaluating Gal-3–targeted binding and immunomodulation, avoiding the artificial context of exogenous Gal-3 supplementation. The elevated Gal-3 expression observed in our M1 model aligns with this inflammatory role, and the low-level expression detected in M0 macrophages aligns with previously reported baseline levels [28]. In Fig. 3H, after incubation with fluorescently labeled MCP or oxMCP, oxMCP exhibited markedly stronger fluorescence signals than MCP, indicating a higher binding affinity. In addition, confocal microscopy revealed that both MCP-FL and oxMCP-FL exhibited spatial colocalization with Gal-3, as evidenced by the appearance of orange and yellow fluorescence in the merged images, supporting their specific binding to Gal-3 under inflammatory conditions. A quantitative analysis further supported this enhancement, with oxMCP showing an approximately 2.4-fold greater signal intensity compared to MCP (Fig. 3J). These findings underscore that the superior biological activity of oxMCP primarily arose from its increased Gal-3-binding affinity, a consequence of its structural and physicochemical transformation.

**Fig. 4:**
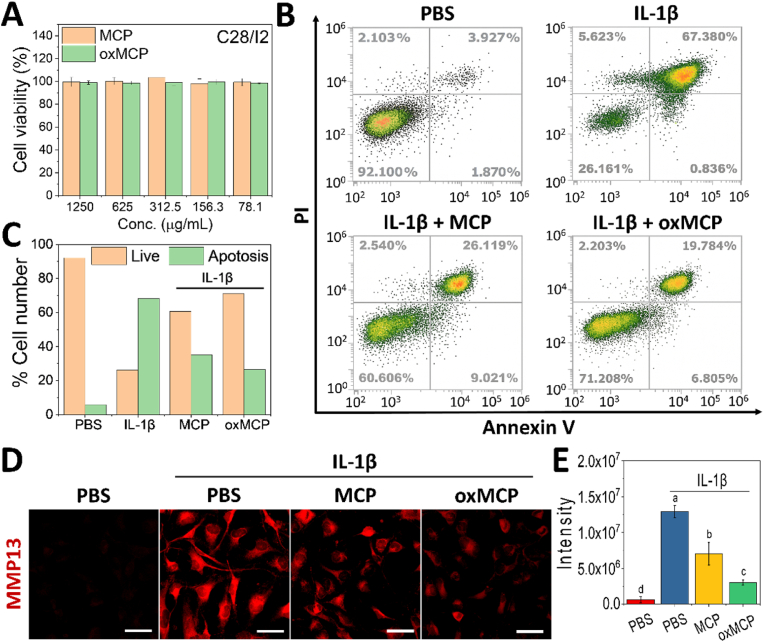
Chondroprotective activity of oxMCP under inflammatory conditions. (A) Cell viability of C28/I2 chondrocytes treated with MCP or oxMCP (*n* = 6). (B, C) Flow cytometric analysis of apoptosis in IL-1β-stimulated chondrocytes (*n* = 3). (D) Immunofluorescence imaging of MMP-13 expression in chondrocytes (scale bars: 50 μm). (E) Quantification of MMP-13 signal intensity using ImageJ software (*n* = 3). Data are presented as the mean ± standard deviation; *p* < 0.05.

Given the central role of Gal-3 in chondrocyte apoptosis and matrix degradation [[13], [14], [15]], we next examined whether the enhanced Gal-3-binding affinity of oxMCP could confer superior chondroprotective effects under inflammatory conditions. The C28/I2 human chondrocyte cell line was used as an *in vitro* model to assess the cytocompatibility and functional responses. As shown in Fig. 4A, oxMCP exhibited excellent biocompatibility across a wide concentration range (78.1–1250 μg/mL), with cell viability consistently above 95 %, comparable to MCP, confirming that chemical modifications of oxMCP did not compromise its compatibility with chondrocytes. To evaluate its protective effects under inflammatory stress, IL-1β was used to induce apoptosis in C28/I2 cells. This model is widely recognized as a relevant *in vitro* paradigm for osteoarthritis, as it recapitulates key pathological features such as oxidative stress, catabolic enzyme induction, and chondrocyte apoptosis [3,4,50]. Importantly, Galectin-3 has been reported to be upregulated in IL-1β–stimulated chondrocytes and to amplify inflammatory signaling through the TLR4/NF-κB and oxidative stress pathways [50,51]. In line with this, previous MCP-based studies using IL-1β–induced chondrocyte models have demonstrated that their observed anti-inflammatory and chondroprotective effects were mediated via Gal-3 blockade [13,14]. Building upon this established framework, we sought to determine whether the structurally reprogrammed oxMCP, with enhanced Gal-3 binding capacity, could further improve chondrocyte protection under this pathophysiological condition. As shown in Fig. 4B and C, IL-1β stimulation markedly increased the apoptotic population to over 65 %. MCP treatment attenuated this effect, while oxMCP provided significantly greater protection, reducing the apoptotic fraction to below 30 %, underscoring its superior anti-apoptotic activity. We further examined MMP-13 expression, a representative marker of ECM degradation. IL-1β strongly upregulated MMP-13 expression, while both MCP and oxMCP suppressed it to various degrees. Notably, oxMCP reduced the MMP-13 signal intensity by 2.3-fold compared to MCP (Fig. 4E). These findings, in line with observed enhancements in Gal-3 binding and M1-suppressive immunomodulatory function, underscore that the superior chondroprotective efficacy of oxMCP is structurally driven and mechanistically coherent.

### 3.3 oxMCP-enabled hydrogel formation and BBR-driven enhancement of gel properties tailored for intra-articular injection

**Fig. 5:**
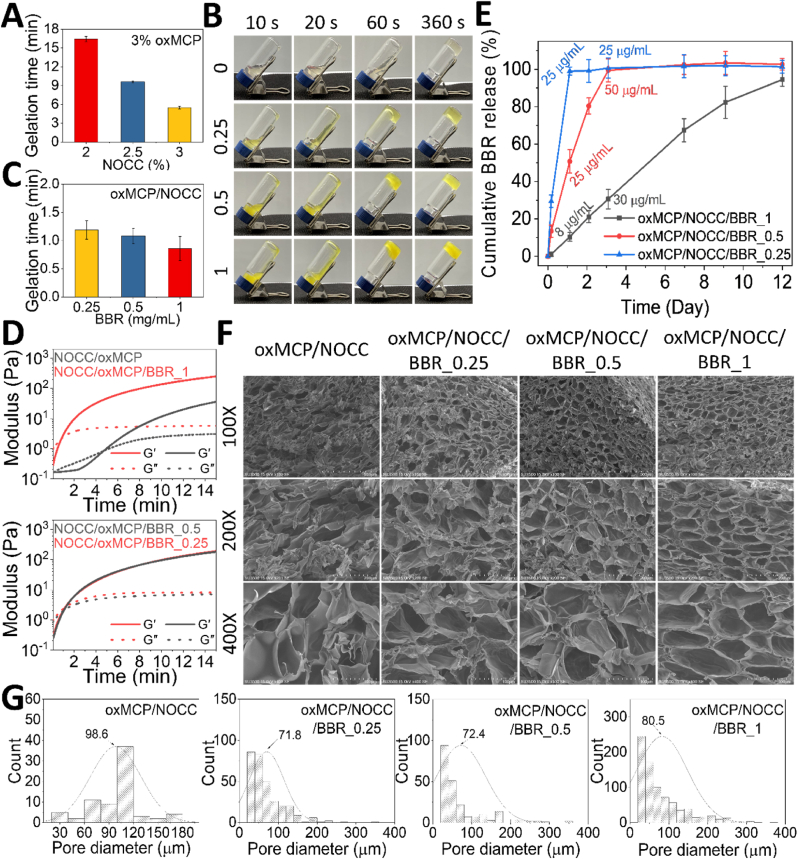
Fabrication and structural reinforcement of oxMCP/NOCC hydrogels by incorporating BBR. Gelation time of oxMCP/NOCC hydrogels with different NOCC (A) and BBR (C) concentrations, determined by the inverted tube method (*n* = 3). (B) Representative inverted tube test showing gelation behavior. (D) Time-resolved gelation profiles measured by rheometry. (E) Cumulative BBR release from hydrogels at pH 7.4 and 37 °C (*n* = 3). (F) SEM images of freeze-dried hydrogels. (G) Pore size distributions of corresponding hydrogels quantified using ImageJ software (*n* = 3).

Beyond its biological activity, oxMCP also demonstrated structural utility as a primary scaffold material for hydrogel fabrication. The aldehyde groups generated via periodate oxidation enabled rapid and reversible Schiff base crosslinking with amino-bearing polymers, offering a robust platform for constructing injectable, biocompatible hydrogels [22,23]. NOCC, a carboxymethylated chitosan derivative retaining a high proportion of primary amines, is water-soluble and exhibited favorable bioactivity, such as a synergistic anti-inflammatory effect with oxMCP (Fig. 3C and D). Accordingly, we evaluated oxMCP as a dynamic network-forming partner for hydrogel fabrication with NOCC. To maximize oxMCP loading in the hydrogel within its solubility limit, a 3 % (w/v) final concentration was used in all formulations. NOCC and oxMCP were mixed at a 1:1 vol ratio under various NOCC concentrations to evaluate the gelation performance. At 1 % and 1.5 % NOCC, no gelation or delayed gelation (>1 h) was observed, indicating insufficient Schiff base reactivity for practical application. In contrast, increasing the NOCC concentration to 2.0 %, 2.5 %, and 3.0 % resulted in respective gelation times of 16.6, 9.6, and 5.5 min (Fig. 5A). Representative gelation behavior of the optimized oxMCP/NOCC formulation is shown in Fig. 5B. These results confirm the successful fabrication of the oxMCP/NOCC hydrogel and demonstrated a concentration-dependent acceleration in gelation kinetics as the available amino content increased.

Despite the effective suppression of M1-type macrophage activation by oxMCP and NOCC (Fig. 3C and D), neither component was able to promote a significant shift toward the M2 phenotype. Given the essential role of M2 polarization in arthritis repair and regeneration [28], we explored whether the incorporating of a bioactive agent could complement the immunomodulatory profile of the oxMCP/NOCC system. BBR, known for its anti-inflammatory and M2-polarizing activities, was also shown to attenuate cartilage degeneration and disease progression in OA models, thereby reinforcing its therapeutic relevance in OA [29]. It was therefore selected as a therapeutic molecule to demonstrate the potential of the hydrogel system for OA treatment. In parallel, BBR can also serve as a representative small-molecule compound to evaluate the hydrogel's drug-loading and release capabilities. BBR was initially incorporated at its maximum aqueous solubility, and oxMCP/NOCC hydrogels containing various BBR concentrations were subsequently prepared to better match therapeutic demands. As shown in Fig. 5C, BBR incorporation significantly accelerated gelation in a concentration-dependent manner. Notably, the formulation containing 1 mg/mL BBR exhibited the fastest gelation time of 0.85 min. The corresponding time-resolved gelation process is visually shown in Fig. 5B. This trend, along with the overall gelation behavior, was further supported by rheological measurements (Fig. 5D), in which BBR-containing hydrogels exhibited faster gelation and higher storage modulus (G′) values compared to the oxMCP/10.13039/100011526NOCC hydrogel, suggesting that BBR incorporation enhanced gel network formation in a concentration-dependent manner. This acceleration in gelation, together with the enhanced mechanical stiffness observed upon BBR incorporation, suggested that BBR may have participated in or promoted crosslinking interactions within the hydrogel matrix. Based on the native molecular structure of BBR, it is plausible that it can engage in hydrogen bonding, electrostatic interactions, hydrophobic interactions, and π–π stacking within the hydrogel matrix, thereby facilitating additional intermolecular interactions with both NOCC and oxMCP, thus accelerating network formation.

Release profiles of BBR from oxMCP/NOCC hydrogels with various BBR concentrations are shown in Fig. 5E. As the BBR loading increased, the release kinetics exhibited a progressively sustained pattern. Notably, the oxMCP/NOCC/BBR_1 formulation (containing 1 mg/mL BBR) achieved complete release by approximately day 9, whereas lower-concentration groups reached saturation within 1–3 days. Despite its higher drug loading, oxMCP/NOCC/BBR_1 exhibited a slower initial burst, releasing only ∼8 μg/mL on day 1. This moderated release profile, which helps mitigate the cytotoxic risk of BBR while prolonging its therapeutic window [20,30], may be attributed to notable intermolecular interactions with the oxMCP/NOCC matrix, enhanced network integrity, and the presence of localized hydrophobic domains that reduce molecular diffusion.

**Fig. 6:**
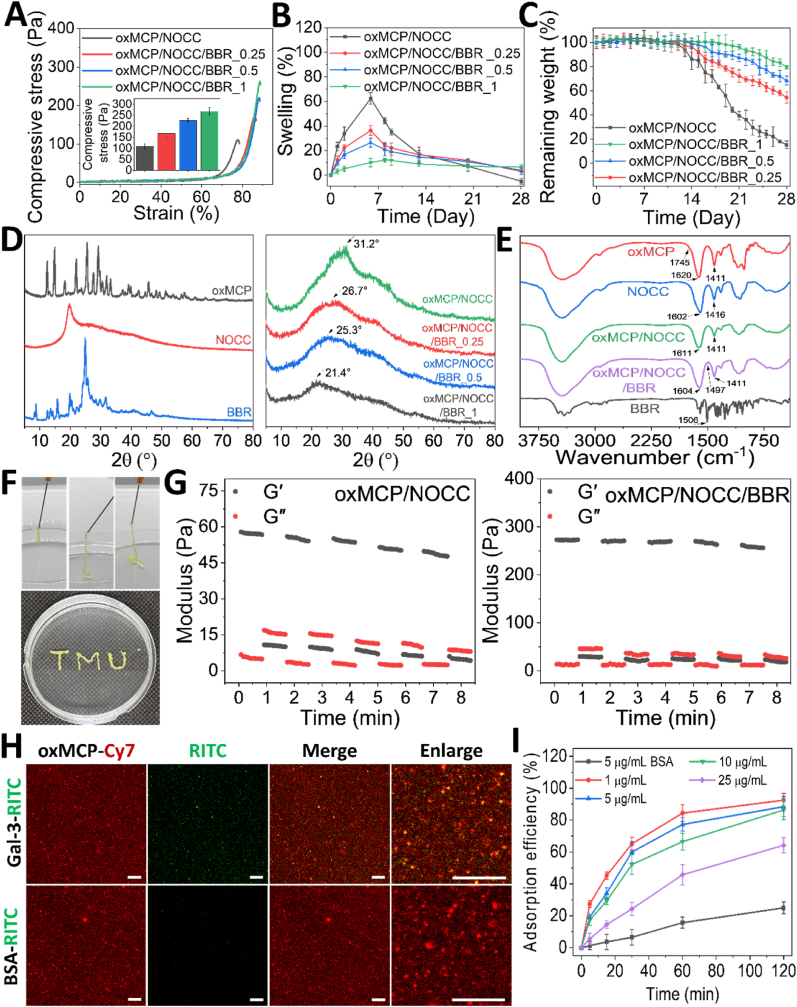
Structural characterization and mechanical, injectable, and degradation properties of oxMCP/NOCC/BBR hydrogels. (A) Texture profile analysis (TPA) of the hydrogel's compressive properties (*n* = 3). (B) Swelling ratios and (C) degradation profiles of hydrogels in RPMI-1640 medium at 37 °C over time (*n* = 3). (D) XRD patterns of oxMCP, NOCC, BBR, and the corresponding hydrogels. (E) FTIR spectra of hydrogel components and composites. (F) Photographs showing hydrogel injectability through a 26 G needle and shape retention after extrusion. (G) Step-strain rheological test assessing the self-healing behaviors of the oxMCP/NOCC and oxMCP/NOCC/BBR hydrogels. (H) Confocal imaging of oxMCP/NOCC/BBR hydrogels labeled with Cy7-oxMCP (red) incubated with RITC-Gal-3 or RITC-BSA (green, 25 μg/mL) at 37 °C for 2 h. **Scale bars: 20 μm.** (I) Gal-3 adsorption kinetics of oxMCP/NOCC/BBR hydrogels at varying concentrations over 2 h.

To further investigate the structural basis underlying the moderated release behavior, SEM imaging and pore size analyses were conducted (Fig. 5F and G). Progressive incorporation of BBR into oxMCP/NOCC hydrogels led to more-robust porous architectures, as evidenced by thicker pore walls and reduced structural collapse. With an increasing BBR concentration, the internal network evolved from loosely connected and irregular pores (oxMCP/NOCC/BBR_0.25), to more continuous and moderately aligned domains (oxMCP/NOCC/BBR_0.5), and finally to a highly organized, honeycomb-like structure in the oxMCP/NOCC/BBR_1 group. Notably, although the oxMCP/NOCC/BBR_1 hydrogel exhibited a slightly larger average pore size (80.5 μm) and more-abundant pores, it maintained a highly coherent and densely interconnected matrix. Compared to hydrogels with lower BBR contents, which showed difficulty forming continuous and well-connected networks, such an architecture is likely to restrict molecular diffusion by increasing path length and reducing permeability, thereby supporting a more-sustained drug release profile (Fig. 5E). These morphological features also indicated enhanced internal cohesion, which was reflected in the increased compressive resistance observed with higher BBR concentrations (Fig. 6A). Consistent with the observed release behavior and structural integrity enhancement, corresponding structural evidence was also observed in swelling and degradation profiles (Fig. 6B and C). In Fig. 6A, all hydrogels exhibited a typical swelling-deswelling trend, with oxMCP/NOCC reaching a peak swelling ratio of 62.5 %. In contrast, the oxMCP/NOCC/BBR_1 hydrogel exhibited minimal swelling, remaining below 10 % throughout the period, likely due to its more-compact network structure and increased hydrophobic character. Degradation profiles similarly showed that the BBR_1 hydrogel had retained over 70 % of its initial weight by day 28, whereas oxMCP/NOCC had rapidly degraded to below 20 % (Fig. 6C). Such low-swelling and slow-degrading properties are highly desirable for intra-articular injections for OA treatment [52], offering a favorable alternative to clinical HA formulations, which are typically degraded within 1–4 weeks post-injection [53].

Taken together, these findings highlight oxMCP as a structurally and functionally versatile material for hydrogel fabrication. The aldehyde-modified backbone enabled dynamic crosslinking with amino-bearing polymers like NOCC to form a stable hydrogel network. The oxMCP/10.13039/100011526NOCC matrix supported the incorporation of small-molecule therapeutics such as BBR. Notably, BBR integration further enhanced the structural cohesion and mechanical robustness of the hydrogel, while contributing to its low-swelling, slow-degrading, and prolonged-release characteristics. Such features are highly desirable for intra-articular injections for OA treatment [20,30,52,53].

### 3.4 Spectroscopic characterization of oxMCP/NOCC/BBR hydrogels

Spectroscopic signatures indicated successful Schiff base crosslinking between oxMCP and NOCC, along with BBR-induced modulation of the hydrogel's molecular architecture. An XRD analysis was conducted to evaluate the intrinsic crystallinity of the hydrogel precursor materials (Fig. 6D). BBR exhibited a series of sharp diffraction peaks at 2θ = 8.7°, 13.4°, 15.8°, 19.8°, 22.2°, and 24.9°, indicative of its highly crystalline nature as a small-molecule isoquinoline alkaloid and reflecting its well-ordered molecular packing in the solid state. In contrast, NOCC exhibited a broad diffuse halo centered at 2θ = 26.3°, indicative of its amorphous nature, as commonly observed in carboxymethylated and partially deacetylated chitosan derivatives. In addition, a sharper reflection at 2θ = 19.7° corresponded to a residual crystalline order originating from the native chitosan structure [20]. oxMCP, as mentioned above, exhibited a set of discrete, sharp reflections across the 2θ range, consistent with partial crystallinity arising from the formation of low-molecular-weight oligosaccharide domains following oxidative backbone cleavage. Upon gelation, both the partial-crystalline signals of oxMCP and residual crystalline reflection of NOCC at 2θ = 19.7° disappeared, and the resulting oxMCP/NOCC hydrogel displayed a broad diffraction feature centered at approximately 2θ = 31.2°, with no sharp peaks remaining. This suggests that dynamic Schiff base crosslinking disrupted the ordered structures of the precursor components and led to a uniform amorphous network. Following BBR incorporation, the hydrogel XRD profiles became progressively flattened, and the primary peak shifted in a concentration-dependent manner from 2θ = 26.7°–25.3° and 21.4°. This gradual low-angle shift reflects an increase in average interchain spacing, likely resulting from BBR intercalation. The absence of new crystalline peaks and the overall loss of ordering suggest that BBR was molecularly dispersed within the hydrogel, where it disrupted polymer chain packing and suppressed the development of ordered regions. These results indicate that the oxMCP/NOCC network provided a structurally compatible matrix that enabled BBR to be uniformly dispersed at the molecular level. The marked disruption of local ordering, with no emergence of new crystalline domains, suggests that BBR formed favorable and balanced intermolecular interactions with the hydrogel components, without causing phase separation or re-aggregation.

Based on integration of release kinetics, and morphological, mechanical, and structural data, the oxMCP/NOCC/BBR_1 hydrogel was selected for subsequent evaluations. Among all formulations, this group not only had the highest BBR-loading capacity but also exhibited the most sustained and prolonged release profile (Fig. 5E), which aligned with its best internal architecture and mechanical integrity (Fig. 5, Fig. 6A). Furthermore, oxMCP/NOCC/BBR_1 showed the lowest swelling ratio and degradation rate (Fig. 6B and C), indicating a highly patient-compliant and stable network suitable for intra-articular injections. The XRD analysis confirmed that even at the highest BBR concentration, the hydrogel maintained complete amorphousness and molecular-level miscibility. Collectively, these findings suggest that oxMCP/NOCC/BBR_1 provided the best-performing platform for controlled drug delivery and structural performance, justifying its use in subsequent *in vitro* and *in vivo* assessments (hereafter referred to as oxMCP/NOCC/BBR).

To further characterize the chemical interactions and network formation within the selected oxMCP/NOCC/BBR_1 hydrogel, FTIR spectroscopy was subsequently employed. In Fig. 6E, NOCC exhibited characteristic absorption bands at 1602 cm^−1^ and 1416 cm^−1^, respectively corresponding to the asymmetric and symmetric stretching vibrations of –COO^-^ groups, indicative of its carboxymethylated chitosan structure [20]. Upon crosslinking with oxMCP, notable spectral changes were observed in the oxMCP/NOCC hydrogel. Most prominently, the aldehyde C=O stretching band at 1745 cm^−1^, present in oxMCP, completely disappeared from the hydrogel spectrum, providing compelling evidence for the successful formation of dynamic imine (C=N) linkages via Schiff base chemistry and confirming the gelation mechanism through dynamic covalent crosslinking [21]. A slight shift in the carboxylate-associated band from 1620 to 1611 cm^−1^ was also observed, possibly reflecting changes in the local chemical environment due to network formation. These observations are indicative of a chemically crosslinked hydrogel structure. Upon BBR incorporation, the asymmetric carboxylate band of the hydrogel further shifted from 1611 to 1604 cm^−1^, indicating the presence of non-covalent interactions such as electrostatic attraction and hydrogen bonding between BBR and functional groups of NOCC and oxMCP. These interactions likely occurred through the quaternary ammonium group and methoxy substituents of BBR, facilitating its anchoring within the polysaccharide matrix. In parallel, the aromatic C=C stretching band of BBR shifted from 1506 to 1497 cm^−1^, which may reflect a reorganization of BBR's intrinsic π–π stacking due to altered molecular packing within the hydrogel environment [20]. These additions or reconstructions of non-covalent interactions likely did not interfere with the primary Schiff base crosslinking, but instead reinforced the polymer network by acting as molecular bridges that enhanced cohesion, stiffness, and sustained drug retention within the hydrogel matrix. Such non-covalent reinforcement mechanisms have also been observed in other systems. For example, halloysite nanotubes, when physically embedded in polysaccharide hydrogels, improved mechanical integrity and enabled sustained drug release via non-covalent interactions with the polymer network [54]. Together, these spectroscopic and structural analyses provide compelling evidence that BBR was molecularly accommodated within the oxMCP/NOCC hydrogel through favorable non-covalent interactions, enabling network reinforcement without compromising chemical crosslinking or structural integrity.

### 3.5 Injectability, self-healing, and Gal-3 sequestration performance of the oxMCP/NOCC/BBR hydrogel

Injectable hydrogels are highly desirable for clinical applications, especially those involving minimally invasive intra-articular procedures. Unlike pre-formed hydrogels that often require surgical implantation, injectable systems enable facile delivery through fine-gauge needles, reducing the surgical burden and improving patient compliance. In Fig. 6F, the injectability of the optimized oxMCP/NOCC/BBR hydrogel was evaluated using a 26 G needle. The hydrogel could be smoothly extruded without clogging or fragmentation, demonstrating excellent flow under shear stress. Upon exiting the needle, the extruded filament maintained its integrity and could form continuous lines or complex shapes, such as patterned lettering (“TMU”), indicating rapid structural recovery and post-injection shape fidelity. This performance is characteristic of reversible dynamic networks, such as Schiff base-crosslinked hydrogels, which were shown to combine shear-thinning flow with self-healing recovery. The ability to extrude through a fine needle while instantly re-solidifying is critical for targeted delivery, conformal filling of irregular anatomical defects, and precision-guided therapies in cartilage repair or sustained drug delivery.

Beyond injectability, the oxMCP/NOCC and oxMCP/NOCC/BBR hydrogels also demonstrated repeatable self-healing behavior, a key feature for biomaterials operating under dynamic mechanical conditions such as intra-articular environments. Self-healing enables hydrogels to recover their mechanical integrity after deformation, contributing to long-term *in vivo* functionality and structural stability. In Fig. 6G, step-strain rheological measurements were conducted by alternating between high strain (500 %) and low strain (1 %) over five consecutive cycles. For the oxMCP/NOCC hydrogel, the storage modulus (G′) exhibited approximately 85 % recovery after the fifth cycle, indicating a decent level of self-healing, albeit with slight attenuation across the cycles. In contrast, the oxMCP/NOCC/BBR hydrogel showed a remarkably stable recovery profile, with approximately 95 % G′ restoration consistently maintained throughout all cycles. This enhancement suggests that the incorporation of BBR reinforced the oxMCP/NOCC network both mechanically and dynamically, by facilitating reversible network reformation, likely through additional non-covalent interactions such as hydrogen bonding or π–π stacking within the hydrogel matrix. These findings indicate that BBR enhanced both the static mechanical strength and the hydrogel's ability to recover from repeated deformation, which is essential for applications involving frequent motion or mechanical impact, such as joint repair or injectable regenerative therapy.

To further verify the Gal-3 sequestration capability of the oxMCP-based hydrogel, dual-fluorescence colocalization analysis was performed by incubating Cy7-labeled oxMCP/NOCC/BBR hydrogels with RITC-labeled Gal-3 or BSA (as a nonspecific control). Confocal imaging revealed extensive spatial overlap between Gal-3-RITC and Cy7-oxMCP signals within the hydrogel matrix, indicating specific affinity-based binding (Fig. 6H). In contrast, BSA-RITC exhibited only weak, scattered fluorescence with minimal overlap, confirming the negligible nonspecific protein adsorption. To quantify this interaction, a time- and concentration-dependent Gal-3 depletion assay was performed by incubating hydrogel discs with RITC-labeled Gal-3 (1–25 μg/mL). As shown in Fig. 6I, adsorption efficiency declined with increasing Gal-3 concentration, consistent with progressive saturation of available binding sites. Over 85 % of Gal-3 was adsorbed within 2 h at lower concentrations (1–5 μg/mL), whereas efficiency decreased to ∼65 % at 25 μg/mL. In contrast, BSA-RITC exhibited consistently low and concentration-independent adsorption, further supporting the high specificity of the oxMCP-Gal-3 interaction. Notably, Gal-3 concentrations in mammalian osteoarthritic synovial fluid have been reported to reach ∼24 ng/mL following cartilage injury [55], a value well below the tested range. These findings highlight the oxMCP hydrogel's ample Gal-3-binding capacity and support its potential to locally scavenge pathological Gal-3 in inflamed joint environments.

### 3.6 Chondroprotective and immunoreprogramming activities of the oxMCP/NOCC/BBR hydrogel

**Fig. 7:**
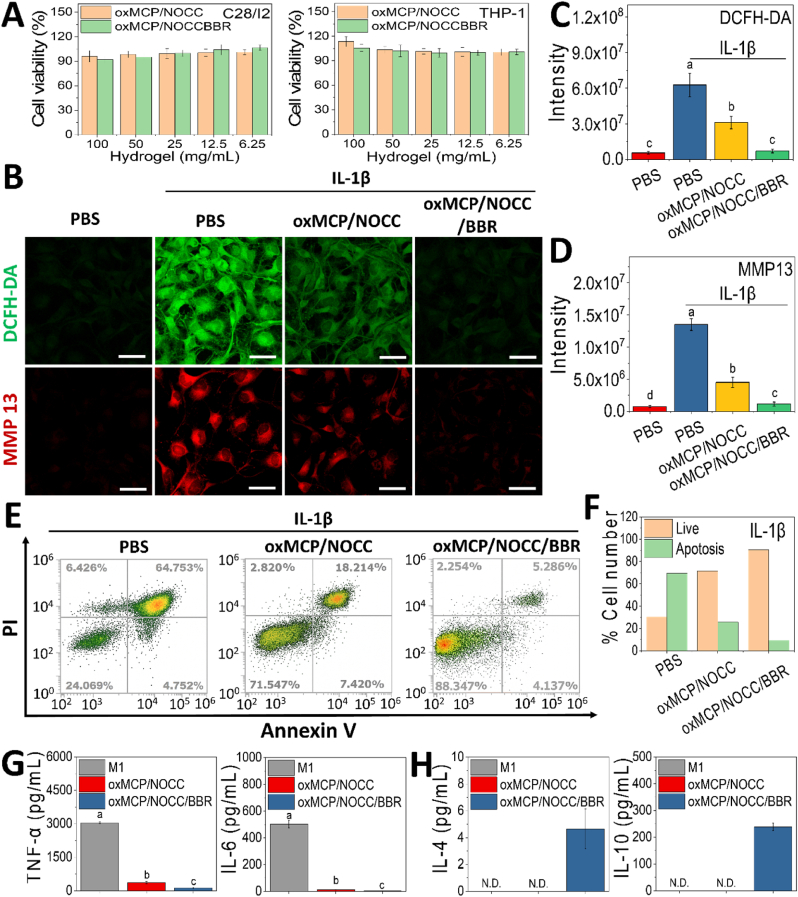
Chondroprotective and immunomodulatory effects of oxMCP/NOCC/BBR hydrogels *in vitro*. (A) Cell viability of C28/I2 chondrocytes and THP-1-derived macrophages treated with various hydrogel formulations (*n* = 6). (B) Confocal imaging of intracellular ROS (DCFH-DA, green) and MMP-13 (red) signals in IL-1β-stimulated chondrocytes (scale bars: 50 μm). (C, D) Quantification of intracellular ROS levels and MMP-13 expression (*n* = 3). (E, F) Flow cytometry analysis of apoptosis in IL-1β–stimulated chondrocytes (*n* = 3). (G, H) Cytokine secretion profiles of M1 (TNF-α and IL-6) and M2 (IL-10 and IL-4) markers from LPS/IFN-γ–stimulated macrophages after treatment (*n* = 6). Data are presented as the mean ± standard deviation; *p* < 0.05.

Building upon the favorable physicochemical and injectable properties of the oxMCP/NOCC/BBR hydrogel, we next investigated its therapeutic potential for OA treatment *in vitro*. Given that oxidative stress, matrix degradation, apoptosis, and dysregulated inflammation are key contributors to OA pathogenesis and are closely modulated by Gal-3 [[13], [14], [15]], we systematically evaluated the chondroprotective capacity of the hydrogel, focusing on its ability to reduce intracellular ROS, suppress MMP-13 expression, and attenuate apoptosis in IL-1β-stimulated chondrocytes. In addition, the hydrogel's immunomodulatory activity was assessed by profiling pro- and anti-inflammatory cytokine expressions and macrophage polarization. Prior to these functional evaluations, the cytocompatibility of oxMCP/NOCC/BBR was assessed by direct co-culture with chondrocytes and macrophages at concentrations ranging 6.25 %–100 % for 2 days (Fig. 7A). Cell viability remained above 90 % under all conditions, with no significant reduction at higher concentrations. These results confirmed the excellent biocompatibility of the hydrogel under direct-contact conditions.

In Fig. 7B and C, IL-1β stimulation markedly increased ROS generation, while treatment with oxMCP/NOCC reduced ROS by approximately 2.0-fold. Although oxMCP likely lost its intrinsic radical-scavenging capacity after periodate oxidation, it exhibited enhanced affinity toward Gal-3 (Fig. 3). The oxMCP/NOCC matrix, enriched in Gal-3-binding domains, may have retained Gal-3-binding bioactivity. Given that oxMCP/NOCC hydrogels are enriched in β-galactoside residues capable of binding Gal-3, the hydrogel likely acts as a “Gal-3-adsorbing sponge” (Fig. 6I). Since Gal-3 is known to contribute to oxidative stress–related inflammatory signaling, including the TLR-4/NADPH oxidase/MAPK pathway [50,51], its sequestration by the hydrogel may suppress ROS production and downstream cytokine expression by disrupting Gal-3-mediated signaling. The modest ROS reduction observed with oxMCP/NOCC thus likely resulted from a combination of Gal-3 interactions and NOCC's inherent antioxidant properties, rather than only through direct radical scavenging. In comparison, oxMCP/NOCC/BBR treatment led to a more-pronounced 8.8-fold reduction in ROS levels. This effect can be primarily attributed to the intrinsic antioxidative capacity of BBR, including direct ROS scavenging and AMPK pathway activation [31], coupled with its well-retained bioactivity under controlled-release conditions (cumulative 21 μg/mL over 48 h, Fig. 5E). The hydrogel likely preserves the bioactivity of BBR under physiological conditions, enabling effective intracellular modulation.

The downstream impact on cartilage matrix metabolism was assessed through MMP-13 expression (Fig. 7D). IL-1β markedly upregulated MMP-13, whereas oxMCP/NOCC treatment reduced its expression by approximately 2.9-fold. This suppression may have resulted from matrix-mediated Gal-3 modulation, consistent with previous findings that MCP mitigates MMP-13 expression and cartilage degradation by disrupting Gal-3–dependent inflammatory and fibrotic signaling [51]. In addition, NOCC may have contributed by downregulating MMP-13 and upregulating tissue inhibitor of metalloproteinase-1 (TIMP-1) in IL-1β–stimulated chondrocytes, potentially through the PI3K/Akt and p38/MAPK signaling pathways [56,57]. The oxMCP/NOCC/BBR hydrogel further suppressed MMP-13 expression by 11.8-fold, restoring it to nearly basal levels, which likely reflects BBR's inhibitory effects on catabolic signaling via NF-κB and MAPK regulation [32]. A similar trend was observed in apoptosis regulation (Fig. 7E and F). IL-1β stimulation induced substantial chondrocyte apoptosis, reaching 71.5 %. This was reduced to 25.6 % following oxMCP/NOCC treatment, and further decreased to 9.4 % with oxMCP/NOCC/BBR treatment. In addition to Gal-3 sequestration, NOCC was reported to protect chondrocytes from IL-1β- or NO-induced apoptosis by restoring the mitochondrial membrane potential [56,58]. BBR was also reported to attenuate apoptosis by inhibiting NF-κB signaling and regulating the Bcl-2/Bax axis [33]. Together, these findings reveal a synergistic chondroprotective effect, wherein the oxMCP/10.13039/100011526NOCC matrix provides a bioactive, galectin-modulating environment, and supports sustained BBR delivery. This integrated system effectively attenuated oxidative stress, suppressed matrix degradation, and preserved chondrocyte viability under osteoarthritic conditions, reinforcing its therapeutic promise in OA management.

Beyond chondrocyte protection, we further examined the immunomodulatory effects of the hydrogel by analyzing macrophage polarization under inflammatory conditions. As shown in Fig. 7G, LPS + IFN-γ induced a pronounced pro-inflammatory M1 phenotype, characterized by elevated levels of TNF-α and IL-6. Treatment with oxMCP/NOCC effectively suppressed both cytokines, indicating that the combined matrix preserved the M1-inhibitory function of oxMCP. However, despite this M1 suppression, oxMCP/NOCC showed a limited capacity to promote M2 polarization, as reflected by minimal expressions of IL-4 and IL-10 (Fig. 7H). This suggests that while the matrix dampens inflammation, it does not actively reprogram macrophages toward a regenerative phenotype. Incorporation of BBR into the hydrogel markedly enhanced M2 polarization from the M1 phenotype, likely via AMPK activation and inhibition of TLR4/NF-κB signaling [59,60]. This shift toward M2 is critical not only for resolving inflammation but also for initiating tissue-regenerative programs essential for cartilage repair. These findings indicate that oxMCP/NOCC provides a Gal-3–modulating, M1-suppressive matrix, and the addition of BBR strategically complements this effect by enabling the M1-to-M2 transition of macrophages.

Collectively, the oxMCP/NOCC/BBR hydrogel demonstrated multifaceted therapeutic potential by simultaneously mitigating oxidative stress, preventing matrix degradation, attenuating apoptosis, and reprogramming macrophage responses. The oxMCP/NOCC matrix acted as a localized Gal-3-adsorbing platform, while BBR synergistically enhanced the system by promoting M2 polarization and reinforcing cellular protection. This cooperative mechanism underpins a robust chondroprotective effect and positions the oxMCP/NOCC/BBR hydrogel as a promising injectable platform for OA intervention.

### 3.7 Therapeutic assessment of the oxMCP/NOCC/BBR hydrogel in OA

**Fig. 8:**
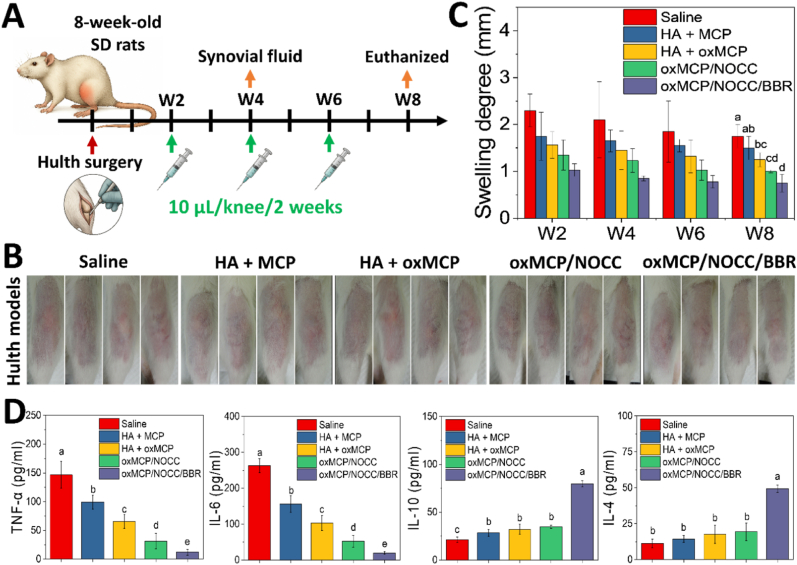
*In vivo* therapeutic evaluation of different formulations in the Hulth-induced OA rat model. (A) Schematic of the experimental timeline showing Hulth surgery, intra-articular injections (10 μL/knee every 2 weeks), and euthanasia at week 8. (B) Representative images of knee joints showing visible swelling. (C) Quantification of knee joint swelling at weeks 2, 4, 6, and 8 (*n = 8*). (D) ELISA quantification of inflammatory cytokines in synovial fluid collected at week 4, including TNF-α, IL-6, IL-10, and IL-4. Data are presented as the mean ± standard deviation (*n = 8*). Different letters indicate statistically significant differences (*p* < 0.05).

To validate the *in vivo* therapeutic performance of the oxMCP-based hydrogel system, a surgically induced OA model was established using the Hulth method, followed by intra-articular administration of test formulations every 2 weeks (three total doses, 10 μL each) as shown in Fig. 8A. Treatment groups were designed to elucidate the stepwise contributions of each formulation component. The HA + MCP group served as a reference control based on a previous report [13], where MCP was delivered via HA to prolong intra-articular residence and enhance Gal-3-targeted anti-inflammatory effects. The HA + oxMCP group was designed to evaluate the benefit of periodate oxidation, which enhances the Gal-3-binding capability. The oxMCP/NOCC group represents a hydrogel formulation with prolonged retention and sustained degradation. The final oxMCP/NOCC/BBR group further integrates BBR, a natural immunomodulator known to promote M2 macrophage polarization, thereby enhancing resolution of inflammation and promoting tissue regeneration.

Representative images of rat knees at week 8 (Fig. 8B) demonstrated that untreated OA joints displayed persistent swelling and erythema, while the HA + MCP and HA + oxMCP groups showed moderate visual improvement. In contrast, the joint appearance in both hydrogel groups was markedly restored. Quantitative analysis of the joint swelling degree (Δ*D*, Fig. 8C) showed that HA + MCP moderately reduced swelling (1.5 ± 0.25 mm), while HA + oxMCP was more effective (1.25 ± 0.15 mm), confirming oxidation-enhanced bioactivity. Transitioning to a hydrogel form further reduced swelling (1.0 ± 0.03 mm), and oxMCP/NOCC/BBR exhibited the most significant effect (0.75 ± 0.18 mm), approaching normal joint dimensions. These findings support that both hydrogel formulation and BBR co-delivery significantly improved the therapeutic efficacy. Cytokine profiling of synovial fluid at week 4 (Fig. 8D) revealed that OA induction markedly increased TNF-α and IL-6 levels. HA + MCP treatment resulted in partial reductions, whereas HA + oxMCP achieved more-pronounced suppression. The oxMCP/NOCC hydrogel further reduced cytokine levels due to its sustained presence and gradual release profile. Notably, oxMCP/NOCC/BBR treatment led to near-complete suppression of pro-inflammatory cytokines. In terms of regenerative immune responses, only the BBR-containing hydrogel markedly elevated the IL-10 and IL-4 anti-inflammatory cytokines, consistent with its M2-promoting effects observed *in vitro* (Fig. 7H).

**Fig. 9:**
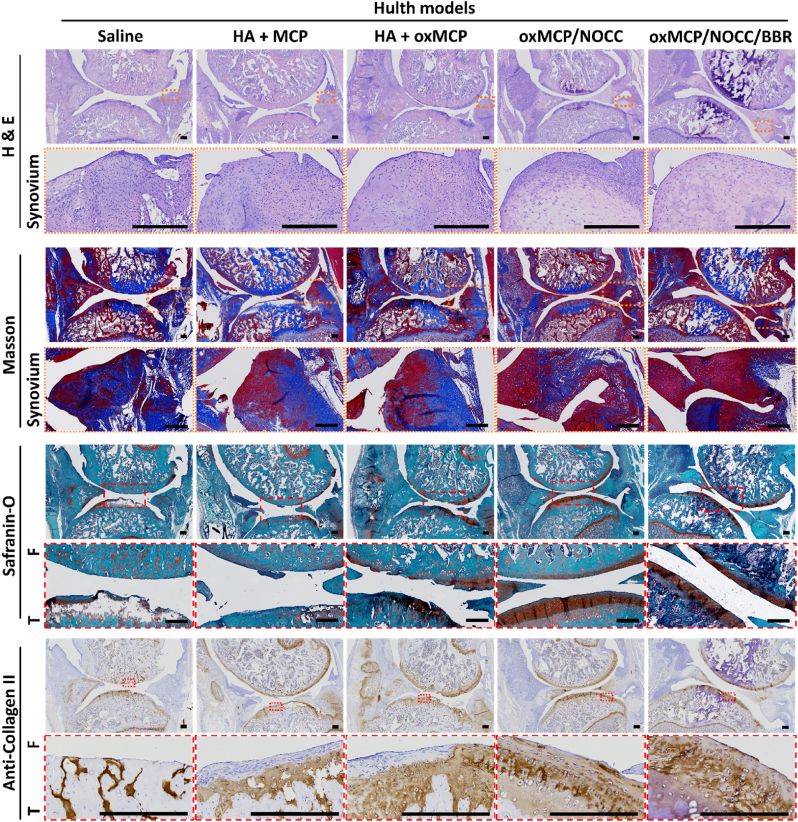
Representative histological and immunohistochemical staining of rat knee joints. H&E staining and Masson's trichrome staining show whole-joint sections and magnified views of the synovium (indicated by orange dashed boxes). Safranin O–Fast Green staining and immunohistochemical staining of type II collagen in whole-joint sections and enlarged views of the femoral (F) and tibial (T) cartilage surfaces (indicated by red dashed boxes). Scale bars: 300 μm.

**Fig. 10:**
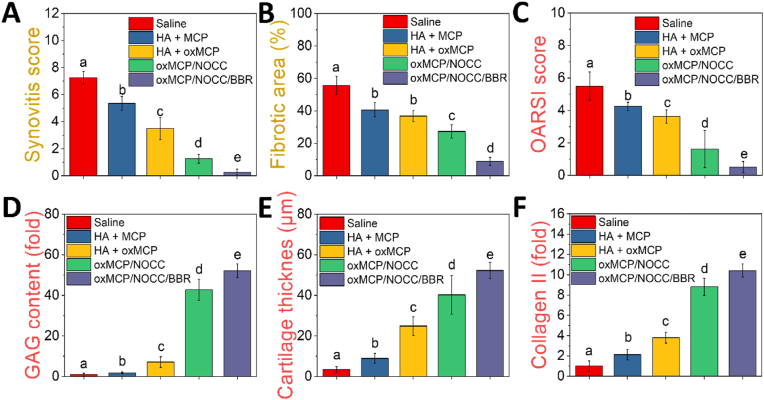
Quantitative histological and immunohistochemical analysis of joint tissues. (A) Synovitis scores and (B) fibrotic areas (%) based on H&E and Masson's trichrome staining, respectively. (C) OARSI score, (D) relative glycosaminoglycan (GAG) content, (E) cartilage thickness, and (F) fold changes of collagen II expression based on Safranin O-Fast Green and collagen II immunohistochemistry. (*n = 4*). Data are presented as the mean ± standard deviation; *p* < 0.05.

In line with these cytokine findings, histological assessment of the synovium was performed at week 8 to further confirm the anti-inflammatory and immunoregulatory effects of the treatments at the tissue level. Given the critical role of chronic synovitis and fibrotic remodeling in perpetuating OA progression, H&E staining and Masson's trichrome staining were conducted to evaluate inflammatory cell infiltration, synovial thickening, and fibrotic matrix deposition across different treatment groups. A histological analysis of the synovium revealed pronounced pathological remodeling in the OA group, including dense inflammatory cell infiltration and a disorganized tissue structure, as shown by H&E staining (Fig. 9). Quantitative synovitis scoring (Table S2) confirmed severe inflammation (7.2 ± 0.5, Fig. 10A), accompanied by extensive subintimal collagen deposition revealed by Masson's trichrome staining, with the fibrotic area reaching 55.7 % ± 5.7 % (Fig. 10B). HA + MCP treatment modestly attenuated both synovitis (5.3 ± 0.6) and fibrosis (40.6 % ± 4.4 %), while HA + oxMCP provided greater improvements (3.5 ± 0.8 and 36.8 ± 3.4 %, respectively), reflecting the enhanced Gal-3 sequestration effect of oxidized MCP. Notably, hydrogel-based delivery further amplified the therapeutic efficacy, with oxMCP/NOCC treatment significantly reducing synovitis (1.3 ± 0.3) and the fibrotic area (27.4 % ± 4.1 %). The most pronounced improvement was observed in the oxMCP/NOCC/BBR group, which exhibited minimal inflammatory infiltration (0.3 ± 0.2) and fibrosis (8.9 % ± 2.5 %). The reduction in synovitis likely resulted from potent M1 macrophage suppression, consistent with downregulation of pro-inflammatory cytokines shown earlier (Fig. 7, Fig. 8D). By attenuating inflammatory signaling, the oxMCP/NOCC/BBR hydrogel effectively disrupted the feedforward loop linking synovial inflammation and fibrotic remodeling [61]. These results demonstrate that the oxidation significantly enhanced the immunoregulatory efficacy of MCP, and that further formulation into a long-retention hydrogel amplified its therapeutic impact *in vivo*. Incorporating of BBR provided an additional anti-inflammatory boost, collectively resulting in superior suppression of synovial inflammation and fibrotic remodeling compared to all other treatments.

Articular cartilage degeneration was assessed with safranin O-Fast Green staining and scored using the OARSI grading system (Table S3). In the OA group, severe cartilage erosion and extensive loss of proteoglycan staining were observed, with an OARSI score of 5.5 ± 0.8 (Fig. 9, Fig. 10C). HA + MCP treatment yielded modest cartilage preservation (4.3 ± 0.3), while HA + oxMCP further improved the joint integrity (3.6 ± 0.4), reflecting the enhanced bioactivity of oxMCP (Fig. 4). Notably, the oxMCP/NOCC hydrogel group showed significant improvement (1.6 ± 1.1), while the oxMCP/NOCC/BBR group exhibited marked restoration of cartilage integrity (0.5 ± 0.3). Quantitative analyses of sulfated glycosaminoglycans (GAGs), critical ECM components responsible for water retention and compressive resistance, further supported these findings. GAG levels progressively increased across the treatment groups, reaching 1.7- (HA + MCP), 7.1- (HA + oxMCP), 42.6- (oxMCP/NOCC), and 52.3-fold (oxMCP/NOCC/BBR) relative to the OA group (Fig. 10D). Cartilage thickness followed a consistent trend, increasing from 3.5 μm (OA) to 8.9, 24.9, 40.2, and 52.3 μm in the respective groups (Fig. 10E). Additionally, immunohistochemical staining for type II collagen, the principal fibrillar protein in hyaline cartilage, revealed a similar pattern. Collagen II expression was minimal in the OA group (1.0-fold), but progressively increased across the treatment groups, reaching 2.1- (HA + MCP), 3.8- (HA + oxMCP), 8.8- (oxMCP/NOCC), and 10.4-fold (oxMCP/NOCC/BBR) relative to the OA controls (Fig. 9, Fig. 10F). These results demonstrated that each level of formulation advancement, polysaccharide structural reprogramming, hydrogel formulation, and BBR incorporation, synergistically contributed to restoring cartilage matrix composition, structural integrity, and ECM protein expression, thereby promoting effective *in vivo* chondral repair.

To rigorously evaluate the therapeutic efficacy, our *in vivo* model was deliberately designed with heightened stringency, involving full-thickness cartilage removal (Hulth surgery rather than ACLT), a reduced injection frequency (once every 2 weeks instead of weekly), and a lower MCP dose per injection. Even under these harsher conditions, the oxMCP/NOCC/BBR hydrogel outperformed the HA + MCP system as reported by Chen et al. [13], demonstrating superior suppression of joint swelling and inflammatory cytokines. Zhang et al. employed a membrane-based MCP delivery strategy applied directly to the joint surface, and achieved immunomodulatory effects under milder OA conditions [14]. He et al. developed MCP-functionalized GelMA/HAMA scaffolds for *in vitro* tissue engineering and partial-thickness defects [17]. In contrast, our system achieved comparable or enhanced therapeutic outcomes across both inflammatory and histological metrics in a full-joint OA model, via injectable administration and with integrated BBR-driven immunomodulation. Beyond inflammatory control, our system also achieved more-pronounced histological improvements than those reports [13,14], including a >50-fold increase in the GAG content and >10-fold upregulation of collagen II expression, along with significantly reduced OARSI and synovitis scores. All three studies confirm MCP's Gal-3-mediated chondroprotective and anti-inflammatory roles; however, our formulation uniquely enabled injectable administration and simultaneous immunomodulation through BBR.

Taken together, these findings underscore the superior *in vivo* performance of the oxMCP/NOCC/BBR hydrogel, driven by rational structural modification of MCP (enhanced bioactivity), formulation optimization (injectable hydrogel with sustained release characteristics), and the synergistic effects introduced by BBR (M2 macrophage polarization and chondrocyte protection). This platform effectively alleviates joint swelling, reprograms synovial inflammation, restores immune homeostasis, and promotes cartilage repair in a stringent OA model, supporting its potential as a localized, disease-modifying strategy for OA therapy.

## 4 Conclusions

This study presents a multifunctional injectable hydrogel system developed by structurally reprogramming MCP into an oxidized form (oxMCP) that serves as both a dynamic crosslinking scaffold and a Gal-3-binding polysaccharide with enhanced biological responsiveness. Upon combining n with NOCC, oxMCP formed a robust, self-healing, and injectable hydrogel network, establishing its potential as a versatile platform for intra-articular applications. Notably, the high-affinity Gal-3-binding capability of oxMCP translated into pronounced therapeutic benefits, including more-effective suppression of M1 macrophage activation and improved protection of chondrocytes from inflammation-induced apoptosis and matrix degradation. The BBR incorporation further enhanced the hydrogel by accelerating gelation, reinforcing the mechanical strength, reducing swelling, delaying degradation, and enabled sustained intra-articular release. In parallel, BBR also endowed the hydrogel with immunoregulatory function by promoting M1-to-M2 macrophage polarization and amplifying chondroprotective effects. Collectively, this work establishes oxMCP as both a bioactive Gal-3-modulating polysaccharide and a structurally tunable hydrogel-forming matrix. Through integration with BBR, the resulting system offers a synergistically engineered, biologically potent, and clinically promising platform for localized, durable, and disease-modifying OA therapy. However, a key limitation should be acknowledged: greener and scalable synthesis routes should be explored to enhance translational feasibility.

Beyond OA, this Gal-3-modulating hydrogel platform may hold translational potential for a broad spectrum of Gal-3-associated disorders. Given Gal-3's pivotal role in fibrosis, tumor progression, and immune dysregulation, this injectable system could be further adapted for site-specific Gal-3 sequestration in diseases such as cardiac fibrosis, idiopathic pulmonary fibrosis, and tumor-associated macrophage-enriched solid tumors. By overcoming formulation limitations of conventional MCP, this structurally tunable, Gal-3-modulating hydrogel lays the groundwork for localized therapies across a spectrum of Gal-3-related pathologies.

## CRediT authorship contribution statement

**Chi Lin:** Writing – original draft, Visualization, Validation, Methodology, Investigation, Formal analysis, Data curation, Conceptualization. **Fwu-Long Mi:** Writing – review & editing, Supervision, Project administration. **Chia-Yun Cha:** Investigation, Formal analysis, Data curation. **Fang-Yu Hsu:** Investigation, Formal analysis, Data curation. **Siti Ayu Ulfadillah:** Formal analysis. **Min-Lang Tsai:** Formal analysis. **Hsien-Tsung Lu:** Writing – review & editing, Supervision, Resources, Project administration, Methodology, Conceptualization.

## Declaration of competing interest

The authors declare that they have no known competing financial interests or personal relationships that could have appeared to influence the work reported in this paper.

## Data Availability

Data will be made available on request.
