# Supplementary material for: Structurally reprogrammed modified citrus pectin (MCP) enables potentiated galectin-3 sequestration and injectable carboxymethyl chitosan/berberine hydrogel construction for osteoarthritis immunotherapy
Source: Mater Today Bio. 2025 Sep 20;35:102330. doi: 10.1016/j.mtbio.2025.102330 (PMC12509201; doi:10.1016/j.mtbio.2025.102330)
Supplement: Multimedia component 1 [file mmc1.docx]

Supporting Information (SI)

**Structurally Reprogrammed Modified Citrus Pectin (MCP) Enables Potentiated Galectin-3 Sequestration and Injectable Carboxymethyl Chitosan/Berberine Hydrogel Construction for Osteoarthritis Immunotherapy**

Chi Lin ^1§^, Fwu-Long Mi ^1,2,3,4§^, Chia-Yun Cha ^2^, Fang-Yu Hsu ^1^, Siti Ayu Ulfadillah ^5, 6^, Min-Lang Tsai ^5^, Hsien-Tsung Lu ^4, 7, 8*^

^1^ Department of Biochemistry and Molecular Cell Biology, School of Medicine, College of Medicine, Taipei Medical University, Taipei City 11031, Taiwan, ROC.

^2^ Graduate Institute of Medical Sciences, College of Medicine, Taipei Medical University, Taipei City 11031, Taiwan, ROC.

^3^ Graduate Institute of Nanomedicine and Medical Engineering, College of Biomedical Engineering, Taipei Medical University, Taipei City 11031, Taiwan, ROC.

^4^ International Ph.D. Program in Cell Therapy and Regenerative Medicine, Taipei Medical University, Taipei City 11031, Taiwan, ROC.

^5^ Department of Food Science, National Taiwan Ocean University, Keelung 202301, Taiwan, ROC.

^6^ Sriwijaya University, Department of Fisheries Product Technology, Agriculture Faculty, Indralaya 30662, South Sumatra, Indonesia.

^7^ Department of Orthopedics, Taipei Medical University Hospital, Taipei City 11031, Taiwan, ROC.

^8^ Department of Orthopedics, School of Medicine, College of Medicine, Taipei Medical University, Taipei City 11031, Taiwan, ROC.

* To whom correspondence should be addressed: Lu7788@ tmu.edu.tw (Dr. Hsien-Tsung Lu)

^§^ Two first authors contributed equally to this work.

**Table. S1** Molecular weight parameters of MCP and oxidized MCP (oxMCP) with different oxidation degrees, determined by gel permeation chromatography (GPC).

|  | oxMCP 0.6 | oxMCP 0.4 | oxMCP 0.2 | oxMCP 0.1 | MCP |
| --- | --- | --- | --- | --- | --- |
| Mp | 2301 | 5630 | 8656 | 9366 | 10436 |
| Mn | 3270 | 5781 | 5676 | 5514 | 12120 |
| Mw | 5086 | 12608 | 11926 | 12660 | 19723 |
| PDI | 1.56 | 2.19 | 2.10 | 2.29 | 1.63 |

Values include peak molecular weight (Mp), number-average molecular weight (Mn), weight-average molecular weight (Mw), and polydispersity index (PDI).

**Table. S2** Synovitis scoring criteria based on H&E-stained sections of rat knee joints.

| Feature | Score 0 | Score 1 | Score 2 | Score 3 |
| --- | --- | --- | --- | --- |
| Synovial lining thickness | 1 cell layer (normal) | 2–3 cell layers | 4–5 layers | ≥6 layers |
| Inflammatory cell infiltration | None | Mild perivascular infiltration | Moderate infiltration into subintima | Dense, diffuse infiltration involving large areas |
| Stromal cellularity / disorganization | Normal cell density and architecture | Slightly increased cellularity | Moderately increased, with focal disorganization | Marked hypercellularity with diffuse stromal disorganization |

^1^ Three histological features, synovial lining hyperplasia, inflammatory cell infiltration, and stromal cellularity, were each scored from 0 to 3 and summed to yield a total synovitis score (maximum = 9).

^2^ Scoring criteria were adapted and modified from the OARSI rat histopathology guidelines [1].

**Table. S3** Histopathological grading criteria for articular cartilage degeneration based on the OARSI scoring system for rat models.

| Score | Criteria |
| --- | --- |
| 0 | Normal cartilage with intact surface and uniform staining |
| 1 | Surface discontinuity (minimal fibrillation) |
| 2 | Vertical clefts within superficial zone |
| 3 | Vertical clefts extending into mid zone |
| 4 | Erosion reaching the calcified cartilage |
| 5 | Denudation of cartilage with deformation of underlying bone |
| 6 | Complete cartilage loss with severe subchondral bone damage |

Cartilage sections were evaluated using Safranin O–Fast Green staining, and scored from 0 (normal) to 6 (severe degeneration with subchondral bone damage), as adapted from the OARSI rat histopathology guidelines [1].

**Reference**

[1] N. Gerwin, A. Bendele, S. Glasson, C. Carlson, The OARSI histopathology initiative–recommendations for histological assessments of osteoarthritis in the rat, Osteoarthritis and cartilage. 18 (2010) S24-S34. <https://doi.org/10.1016/j.joca.2010.05.030>.
